# IGFBP2 modulates the chemoresistant phenotype in esophageal adenocarcinoma

**DOI:** 10.18632/oncotarget.4532

**Published:** 2015-07-17

**Authors:** Amy L. Myers, Lin Lin, Derek J. Nancarrow, Zhuwen Wang, Daysha Ferrer-Torres, Dafydd G. Thomas, Mark B. Orringer, Jules Lin, Rishindra M. Reddy, David G. Beer, Andrew C. Chang

**Affiliations:** ^1^ Department of Surgery, University of Michigan, Ann Arbor, MI, USA; ^2^ Department of Pathology, University of Michigan, Ann Arbor, MI, USA

**Keywords:** IGFBP2, esophageal cancer, chemotherapy resistance, ERK, AKT

## Abstract

Esophageal adenocarcinoma (EAC) patients commonly present with advanced stage disease and demonstrate resistance to therapy, with response rates below 40%. Understanding the molecular mechanisms of resistance is crucial for improvement of clinical outcomes. IGFBP2 is a member of the IGFBP family of proteins that has been reported to modulate both IGF and integrin signaling and is a mediator of cell growth, invasion and resistance in other tumor types. In this study, high *IGFBP2* expression was observed in a subset of primary EACs and was found to be significantly higher in patients with shorter disease-free intervals as well as in treatment-resistant EACs as compared to chemonaive EACs. Modulation of IGFBP2 expression in EAC cell lines promoted cell proliferation, migration and invasion, implicating a role in the metastatic potential of these cells. Additionally, knockdown of IGFBP2 sensitized EAC cells to cisplatin in a serum-dependent manner. Further *in vitro* exploration into this chemosensitization implicated both the AKT and ERK pathways. Silencing of IGFBP2 enhanced IGF1-induced immediate activation of AKT and reduced cisplatin-induced ERK activation. Addition of MEK1/2 (selumetinib or trametinib) or AKT (AKT Inhibitor VIII) inhibitors enhanced si*IGFBP2*-induced sensitization of EAC cells to cisplatin. These results suggest that targeted inhibition of IGFBP2 alone or together with either the MAPK or PI3K/AKT signaling pathway in IGFBP2-overexpressing EAC tumors may be an effective approach for sensitizing resistant EACs to standard neoadjuvant chemotherapy.

## INTRODUCTION

The incidence of esophageal adenocarcinoma has been increasing steadily in industrialized countries over the past four decades and continues to rise [1]. Despite advances in endoscopic surveillance and multimodality treatment regimens, the prognosis for EAC patients remains poor, with an overall 5-year relative survival rate of less than 20% [2]. While understanding of the progression from Barrett's esophagus to EAC has improved, patients commonly present with advanced stage disease and demonstrate resistance to conventional therapy, with complete response rates to trimodality therapy (chemotherapy, radiation therapy and esophageal resection) below 40% [3–5]. Only complete responders (those patients who are restaged as T0N0 or T1N0) have improved survival as compared to incomplete or non-responders. Understanding the molecular mechanisms for chemoresistance is crucial in order to identify strategies to address such resistance, and thus improve clinical outcome.

The insulin growth factor-1 (IGF1) and IGF1 receptor (IGF1R) signaling pathway, implicated in esophageal cancer growth and progression, has been shown to play pivotal roles in cell growth, differentiation, survival, transformation and metastasis [6–9]. Overexpression of IGF1R is prevalent in esophageal adenocarcinoma (EAC), as high as 75% [10], and has been associated with the progression of Barrett's to EAC [8]. Overexpression of IGF1R also appears to be an independent predictor of survival [10]. Serum IGF-1 level correlates with pathological stage, depth of invasion and prognosis in esophageal cancer patients [11]. Additionally, Doyle et al. (2011) reported a dose-dependent increase in proliferation in response to IGF1 in EAC cells and significantly higher serum IGF1 levels in EAC patients compared to those with Barrett's esophagus or controls [12]. Function-altering polymorphisms in IGF1R [13] as well as IGF1 microsatellite repeats and single strand nucleotide polymorphisms [14, 15] have been shown to affect risk for developing Barrett's esophagus and/or EAC.

Insulin-like growth factor (IGF) binding protein 2 (IGFBP2) is a member of the IGFBP family of proteins that binds both IGF1 and IGF2 in circulation with higher affinity than ligand-receptor interactions and can inhibit or stimulate the growth-promoting effects of IGFs by altering their bioavailability, distribution, stability and/or interactions with cell surface receptors [6, 16–18]. IGFBP2 also contains arginine-glycine-aspartic acid (RGD) and heparin-binding motifs that bind to integrins and extracellular matrix, respectively, to mediate its role in cell detachment, migration and invasion [17–21].

Increased IGFBP2 expression has been reported in many malignancies and has been linked to chemoresistance in ovarian cancer [22], breast cancer [23–25], colon cancer [26], lung cancer [27–29], prostate cancer [30, 31], glioma [32–34] and leukemia [35–37]. It has been shown to be a key player in the development and progression of both glioma [32] and prostate cancer [30]. Inhibition of its expression has been reported to increase apoptosis and decrease migration of human leukemia cells [35], while its overexpression significantly increased the invasive capability of glioblastoma [33, 34] and ovarian cancer cells [38].

Because IGFBP2 has been shown to modulate both the IGF and integrin pathways and is a mediator of cell growth, invasion, and resistance in other tumor types [22–37, 39, 40], we evaluated the functional role of IGFBP2 in EAC progression and chemoresistance. We show that modulation of IGFBP2 expression affects proliferation, motility, and chemosensitization of EAC cells in a serum-dependent manner. Silencing of IGFBP2 affects both AKT and ERK activity and addition of targeted pharmacologic inhibitors of these pathways enhances si*IGFBP2*-induced cisplatin (CDDP) chemosensitization. IGFBP2 is a potential mediator of chemoresistance in a subset of EACs and its modulation in overexpressing EAC tumors may be an effective approach to sensitizing resistant tumors to standard of care chemotherapy.

## RESULTS

### Differential expression of *IGFBP2* in human esophageal tissues

**Figure 1:**
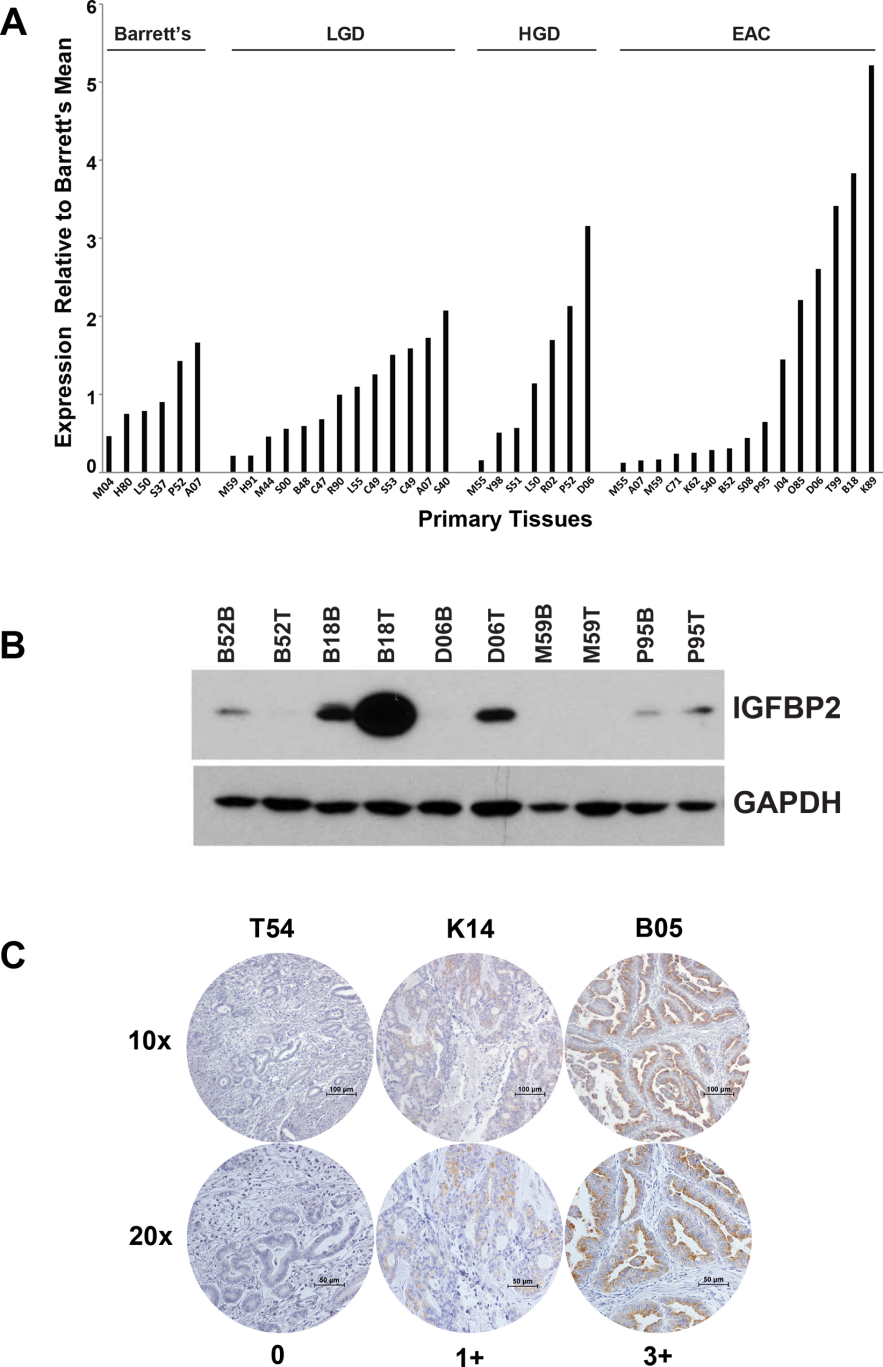
IGFBP2 expression in esophageal tissues and EACs **A.** Affymetrix HG-U133A oligonucleotide microarray of *IGFBP2* expression (K_202718_at) in a progression series of human esophageal tissues ranging from Barrett's metaplasia, low-grade dysplasia, high-grade dysplasia, to adenocarcinoma relative to the Barrett's metaplasia mean; **B.** Immunoblot analysis of protein extracts from 5 micro-arrayed EACs paired with their matching Barrett's metaplasia; **C.** Representative immunohistochemistry of EAC tissue microarrays at both 10× and 20× magnification (0, no staining; 1+, < 10% staining; 2+, 10–50% staining; 3+, > 50% staining).

*IGFBP2* expression levels in a progression series of human esophageal tissues including Barrett's metaplasia, low-grade dysplasia, high-grade dysplasia and EAC were examined by Affymetrix HG-U133A oligonucleotide microarray (Figure 1A). Although many EACs expressed very low levels of *IGFBP2*, a subset of primary tumors expressed higher levels of *IGFBP2*. Expression values for this cohort were confirmed by qRT-PCR (R^2^ = 0.80) (Supplementary Figure S1A). We analyzed the association between *IGFBP2* expression and stage, node status or differentiation. None of these clinical parameters were associated with *IGFBP2* expression (*p* = 1.00, 1.00 or 0.57, respectively, using Fisher's exact test). Immunoblot analysis of protein extracts from 5 paired samples of esophageal adenocarcinoma and associated Barrett's metaplasia indicated increased expression in three of five tumor samples and decreased expression in one of five tumor samples relative to Barrett's metaplasia (Figure 1B). Variable IGFBP2 protein expression was observed among the EAC tissue samples and correlated highly with *IGFBP2* tissue mRNA expression, as noted by both oligonucleotide microarray and qRT-PCR. Immunohistochemistry of tissue microarrays confirmed variable levels of IGFBP2 expression in EACs, ranging from undetectable to high expression at the brush border of several patient sections (Figure 1C). Expression was detected in approximately 23% of tumors but did not correlate with pathologic tumor stage.

**Figure 2:**
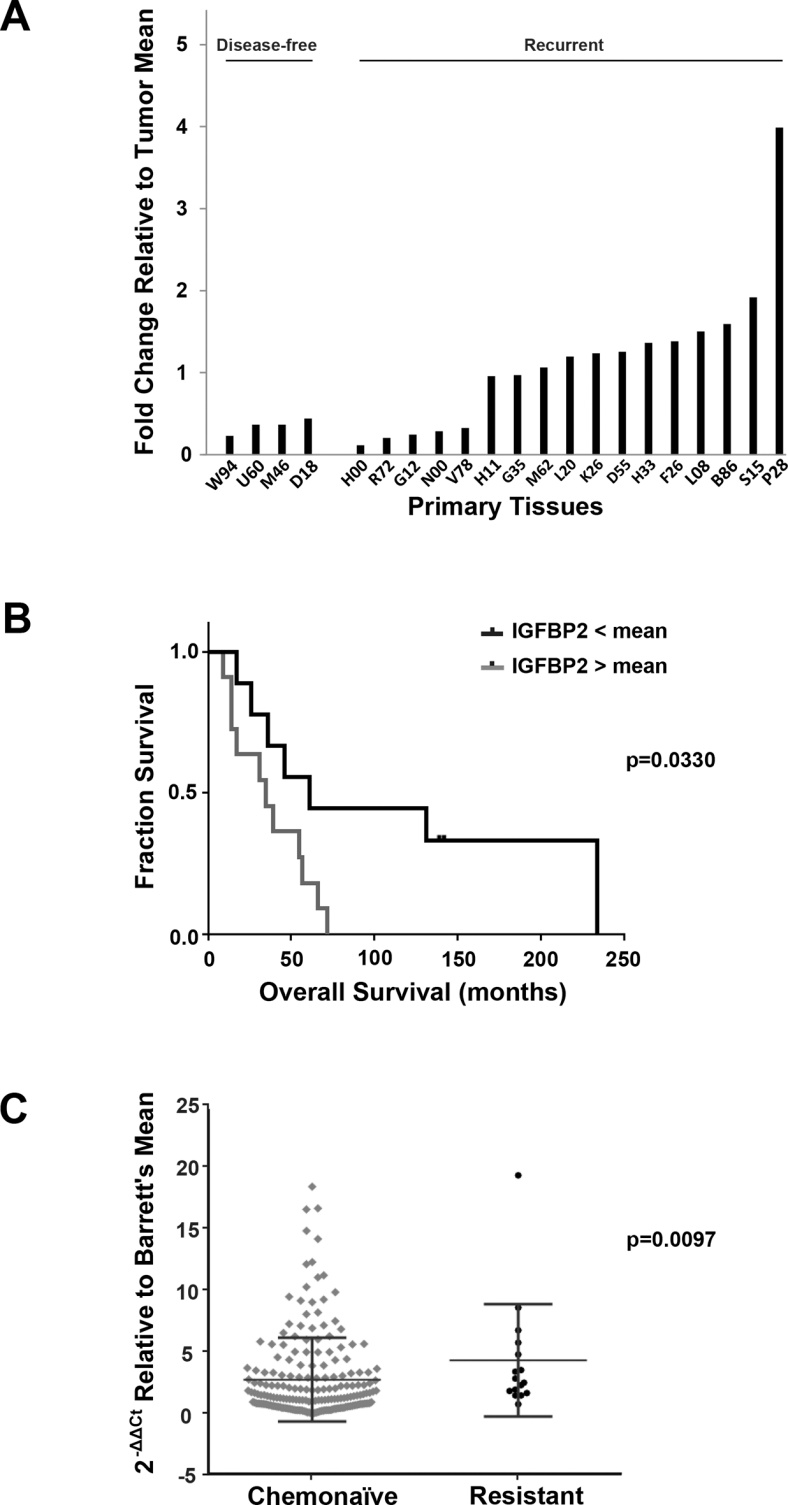
Association of IGFBP2 expression with chemoresistance in EACs **A.** Affymetrix HG-U133A oligonucleotide microarray of *IGFBP2* expression (K_202718_at) in a cohort of disease-free versus recurrent disease chemonaïve EACs relative to the overall tumor mean; **B.** Kaplan-Meier Survival curve of *IGFBP2* expression in this cohort of chemonaïve EACs, grouped according to high (*n* = 11) and low (*n* = 9) IGFBP2 expression relative to the mean (*p* = 0.0330, log rank test); **C.** Box plot representation of real-time PCR analysis of *IGFBP2* expression in 200 chemonaïve EACs and 16 treatment-resistant EACs relative to Barrett's esophagus mean and normalized to *GAPDH*. Differences in *IGFBP2* levels were statistically significant as determined by nonparametric Mann-Whitney analysis (*p* = 0.0097) and categorical binary analysis using a 1.5-fold threshold (*p* = 0.006).

Affymetrix HG-U133A oligonucleotide microarray analysis of a second cohort of twenty EACs, obtained from esophagectomy patients who subsequently received chemotherapy, identified *IGFBP2* as one of the genes with lower expression among disease-free patients as compared to patients with recurrent disease (Figure 2A). Expression levels for this cohort were confirmed by qRT-PCR (R^2^ = 0.80) (Supplementary Figure S1B). In this cohort, patients with higher *IGFBP2* expression had worse overall survival, even when controlled for cancer stage (*p* = 0.033, log rank test) (Figure 2B). Multivariable Cox analyses were performed for stage, node positivity and differentiation. Stage and node positivity were correlated (concordance = 0.703, R^2^ = 0.292), and both of these variables were significantly correlated to overall survival (*p* = 0.014 and 0.0317, respectively). There was no concordance between stage and *IGFBP2* expression, and the IGFBP2 correlation to overall survival was stronger than stage (*p* = 0.0069). Differentiation was not significant due to lack of power (only 8 of 20 samples had known differentiation status).

To address the hypothesis that IGFBP2 is involved in chemoresistance, we performed real-time PCR on 200 EAC samples obtained from previously untreated patients (“chemonaïve”) and 16 treatment-resistant EAC samples to compare their levels of *IGFBP2* expression relative to Barrett's esophagus and normalized to *GAPDH*. Baseline clinical data on all subjects included in this study are provided in Table 1, and chemoradiation therapy regimens for treated patients are detailed in Supplementary Table S1. Although there was considerable variability within the two groups, *IGFBP2* expression was significantly higher in resistant EACs as determined by nonparametric Mann-Whitney analysis (*p* = 0.0097) (Figure 2C). A categorical binary analysis was also performed using a 1.5-fold threshold, which would be equivalent to a 2-fold change assuming approximately 70% tumor content for each tissue analyzed. Expression of *IGFBP2* differed significantly between untreated and treated samples (*p* = 0.006).

**Table 1:** Clinical Characteristics

	Chemonaïve		Resistant	*p*-value
	Surgery only	Adjuvant	Neoadjuvant	
	(*n* = 138)	(*n* = 62)	(*n* = 16)	
**Age, years**				
Median	72	67	63	0.004
Range	25–89	45–84	51–71	
**Gender**				
Male	109(79.0%)	58(93.5%)	16(100.0%)	0.078
Female	29(21.0%)	4(6.5%)	0(0.0%)	
**Smoking history**				
Nonsmoker	12(14.6%)	10(25.6%)	2(13.3%)	0.643
Former	61(74.4%)	28(71.8%)	10(66.7%)	
Current	9(11.0%)	1(2.6%)	3(20.0%)	
Smokeless	1	0	1	
Unknown	55	23	0	
**Disease stage**				
I	37(26.8%)	6(9.7%)	3(18.8%)	0.674
IIA	23(16.7%)	4(6.5%)	1(6.3%)	
IIB	16(11.6%)	10(16.1%)	3(18.8%)	
III	52(37.7%)	39(62.9%)	8(50.0%)	
IV	10(7.2%)	3(4.8%)	1(6.3%)	
**Tumor Grade**				
Well	16(13.8%)	4(8.3%)	1(10.0%)	0.193
Moderate	36(31.0%)	14(29.2%)	6(60.0%)	
Poor	64(55.2%)	30(62.5%)	3(30.0%)	
Unknown	22	14	6	
**Node status**				
No	63(45.7%)	9(14.8%)	7(43.8%)	0.547
Yes	75(54.3%)	52(85.2%)	9(56.3%)	
**Treatment regimen**				
Surgery only	138(100.0%)	0(0.0%)	0(0.0%)	ND
Surgery + CT	0(0.0%)	15(24.2%)	0(0.0%)	
Surgery + RT	0(0.0%)	12(19.4%)	0(0.0%)	
Surgery + CT + RT	0(0.0%)	35(56.5%)	16(100.0%)	
**Recurrence**				
No	67(48.6%)	16(25.8%)	5(31.3%)	0.423
Yes	71(51.4%)	46(74.2%)	11(68.8%)	
**RFS, months**				
Median	26	18	17	0.335
Range	2–253	3–234	7–107	
**Patient status at last contact**				
Alive	29(21.0%)	9(14.5%)	6(37.5%)	0.078
Deceased	109(79.0%)	53(85.5%)	10(62.5%)	
**OS, months**				
Median	28	26	30	0.825
Range	2–253	5–234	7–107	

Abbreviations: RFS, relapse-free survival: OS, overall survival: CT, chemotherapy: RT, radiotherapy: ND, not done

### IGFBP2 expression in EAC cell lines

The three EAC cell lines Flo-1, OE19 and OE33 were utilized in functional assays to investigate the role of IGFBP2 in EAC tumorigenesis and chemoresistance. Real-time PCR analysis of the EAC cell lines Flo-1, OE19 and OE33 revealed high *IGFBP2* expression in Flo-1 cells but minimal expression in OE19 and OE33 cells (Supplementary Figure S2A). Although IGFBP2 protein expression was consistent with the levels of *IGFBP2* mRNA expression in Flo-1 and OE33, OE19 cells expressed relatively high levels of IGFBP2 protein, suggesting a potential post-transcriptional or post-translational modification to stabilize the protein (Supplementary Figure S2B).

### Effect of IGF1 and IGFBP2 on proliferation of EAC cells

Since IGFBP2 may rely on its interaction with IGFs to exact its role in chemoresistance, we evaluated the proliferative effects of exogenous human recombinant IGFs in EAC cell lines. IGF1 promoted dose-dependent growth in Flo-1 and OE33 cells in serum-free conditions but had little to no effect in serum-replete conditions (Supplementary Figure S3A & S3B), suggesting that the proliferative impact of IGF1 is masked in serum-replete conditions. In contrast to what has been previously reported [12], OE19 cells did not significantly respond to IGF1 in the presence or absence of serum (Supplementary Figure S3C), emphasizing its self-sufficiency in growth signaling. Additionally, the proliferative response to exogenous IGF1 was blunted in Flo-1 cells treated with siRNA to *IGFBP2* in the absence of serum (data not shown), suggesting that IGFBP2 may potentiate IGF1-dependent proliferation in these cells.

Treatment with si*IGFBP2* alone reduced proliferation of Flo-1 cells in serum-free conditions, suggesting an IGF-independent role of IGFBP2 in the proliferation of these cells. In most instances, this IGFBP2 effect was seen in the presence or absence of IGFs, but addition of IGFs also enhanced this reduction, possibly due to increased proliferation in IGFBP2-expressing controls. This inhibitory effect of IGFBP2 knockdown was not consistently observed in serum-replete conditions, suggesting that IGFBP2 may have differential roles or significance based on the availability of additional growth factors.

An IGFBP2 expression construct was transfected into OE33 EAC cells with baseline endogenous IGFBP2 expression and stable cells derived from single cell clones were selected for their increased IGFBP2 expression levels (Supplementary Figure S4A). Differential clonal proliferation was observed among the individual OE33/IGFBP2 cells, independent of their IGFBP2 expression levels (Supplementary Figure S4B), indicating that these increased levels of IGFBP2 expression were not responsible for the altered proliferative potential and that an inherent heterogeneity exists in OE33 cells. We did, however, observe a statistically significant proliferative advantage for OE33 cells treated with exogenous human recombinant IGFBP2 in an IGF1-dependent manner in serum-replete conditions. This effect was not observed in serum-free cultures (Supplementary Figure S4C).

### Correlation of *IGFBP2* and EMT-related gene expression

SiRNA knockdown of *IGFBP2* in Flo-1 was associated with increased expression of several epithelial-mesenchymal transition-related genes, including *SNAI1*, *ZEB1*, *VIM*, *MMP9*, and *MMP1* (Supplementary Figure S5A). Treatment with CDDP resulted in a marked increase in *SNAI1*, *SNAI2* and *FN1* expression while *CDH1* and *VIM* expression decreased (Supplementary Figure S5B). *CDH1* levels decreased while *CDH2* levels increased in si*IGFBP2*/CDDP-treated Flo-1 cells as compared to mock-treated or siNon-targeting controls (Supplementary Figure S5C), indicating an “E-cadherin to N-cadherin switch” that has been previously shown to contribute to tumor cell motility and invasion [41, 42]. Expression of *MMP1*, *MMP9* and *SNAI1* was higher in si*IGFBP2*-treated cells as compared to the non-targeting siRNA control, suggesting a potential relationship between IGFBP2 and these genes in CDDP-treated cells (Supplementary Figure S5B).

### IGFBP2-mediated migratory and invasive capacity of EAC cells

To determine the role of IGFBP2 in EAC cell motility, we used si*IGFBP2*-treated Flo-1 cells and IGFBP2 stably-transfected OE33 cells in wound healing assays. IGFBP2-expressing OE33 stable cells migrated further than empty vector controls by 8 hours post-wound and fully closed the wound by 20 hours (Supplementary Figure S6A). Conversely, Flo-1 cells treated with si*IGFBP2* appeared to migrate into the wound more slowly than the non-targeting siRNA control (Supplementary Figure S6B). Boyden chamber assays using IGFBP2-expressing Flo-1 cells showed only marginal Matrigel invasion differences upon knockdown of IGFBP2 (Supplementary Figure S6C). These subtle differences suggest that although IGFBP2 might not be the driver gene for metastasis, this protein does contribute to EAC cell migration and invasion.

### Role of IGFBP2 in chemoresistance *in vitro*

**Figure 3:**
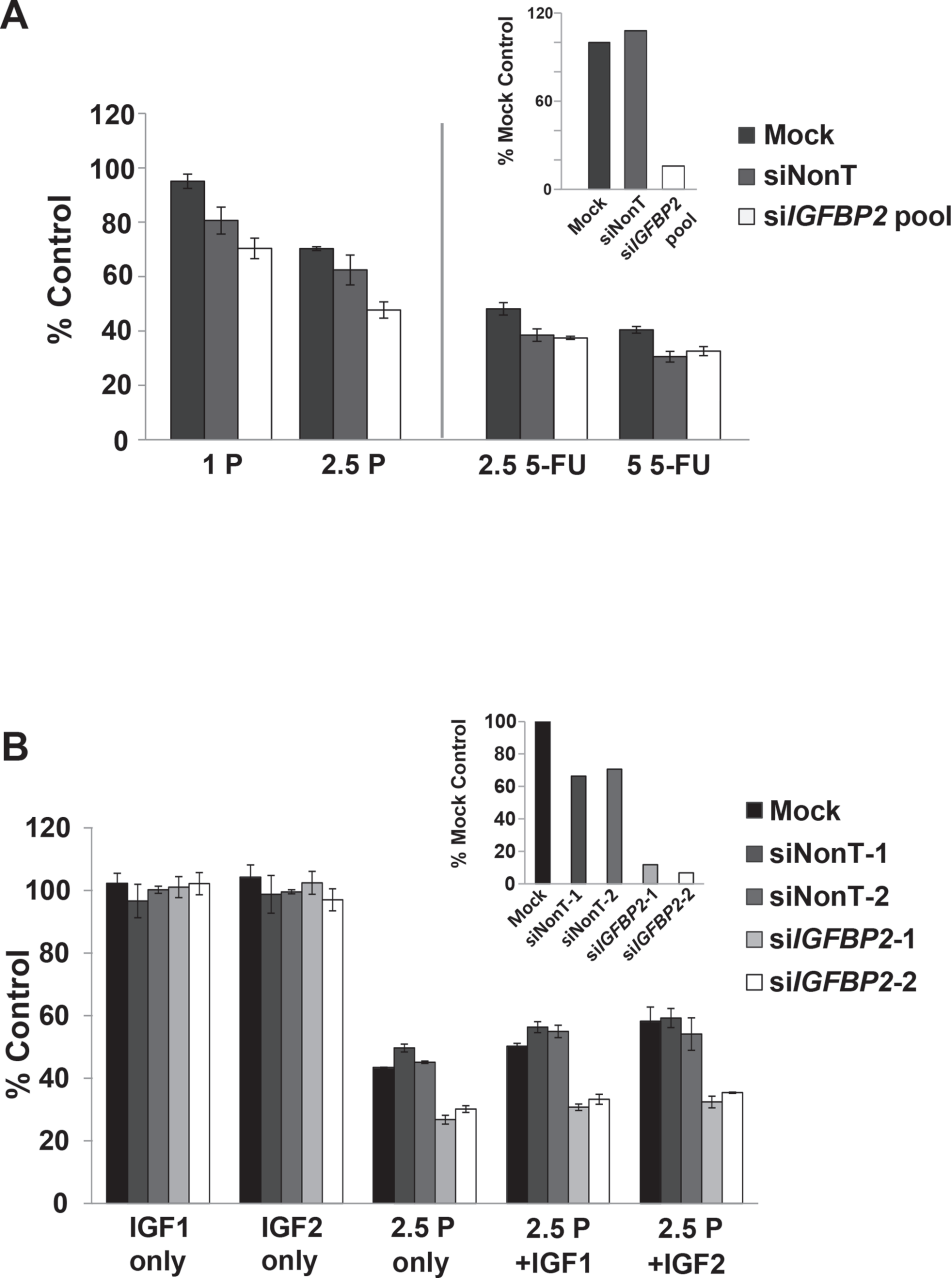
Effect of IGFBP2 modulation on chemosensitivity **A.** Following 24-hour treatment with ON-TARGETplus *IGFBP2* siRNA SMARTpool (si*IGFBP2* pool), siNon-Targeting control (siNonT) or lipofectamine alone (Mock), Flo-1 cells were mock-treated or treated with 1 μg/mL (3.3 μM) CDDP, 2.5 μg/mL (8.3 μM) CDDP, 2.5 μg/mL (19.2 μM) 5-FU or 5 μg/mL (38.4 μM) 5-FU for 3 days and analyzed for cell viability using Cell Proliferation Reagent WST-1. Concurrent qRT-PCR was performed to verify IGFBP2 knockdown. **B.** Following 24-hour treatment with individual ON-TARGETplus *IGFBP2* siRNAs, siNon-Targeting controls or lipofectamine alone, Flo-1 cells were mock-pretreated or pretreated with 200 ng/mL IGF1 or IGF2 for 1 hour followed by mock-treatment or treatment with 1 or 2.5 μg/mL (3.3 or 8.3 μM) CDDP in serum-free or 10% serum DMEM for 3 days. Cell viability was analyzed using Cell Proliferation Reagent WST-1. Columns and error bars are the mean ± SD of 3 or more wells in each experiment. Concurrent qRT-PCR was performed to verify IGFBP2 knockdown. (1 P, 1 μg/mL CDDP; 2.5 P, 2.5 μg/mL CDDP; 2.5 5-FU, 2.5 μg/mL 5-FU; 5 5-FU, 5 μg/mL 5-FU)

In order to evaluate the role of IGFBP2 as a potential modulator of chemoresistance *in vitro*, IGFBP2-expressing Flo-1 cells were pretreated with siRNA to *IGFBP2* while baseline IGFBP2-expressing OE33 cells were transfected with a pEGFP-IGFBP2 expression construct followed by a 3-day treatment with low doses of CDDP or 5-FU. The dose ranges utilized in these assays were clinically relevant as determined by previously reported achievable *in vivo* drug plasma concentrations [43, 44]. Inhibition of IGFBP2 expression sensitized Flo-1 cells to CDDP but not 5-FU in serum-replete conditions (Figure 3A). Addition of either exogenous IGF1 or IGF2 did not appreciably alter this effect (Figure 3B). In serum-free cell cultures, IGFBP2 knockdown did not enhance the response to chemotherapy.

Acute 24 hr treatments with high doses of CDDP following IGFBP2 knockdown were also performed. In contrast to 3-day low-dose CDDP treatments, si*IGFBP2*-treated Flo-1 cells were only slightly sensitized to CDDP (Supplementary Figure S7A), suggesting that the chemosensitization effect of IGFBP2 knockdown may be dependent on its modulation of proliferation and would require longer incubation periods to detect differences. Neither stable overexpression of IGFBP2 (Supplementary Figure S7B) nor exogenous IGFBP2 (Supplementary Figure S7C) altered the chemotherapy sensitivity of OE33 cells, suggesting that this *ERBB2*-amplified [45] cell line is not dependent on IGFBP2 expression for cell survival. The chemosensitization effect of knocking down IGFBP2 was also examined in endogenously-expressing OE19 cells. This more CDDP-resistant EAC cell line also has *ERBB2* amplification [45] and was not as responsive to si*IGFBP2* treatment as Flo-1 cells (Supplementary Figure S7D), suggesting that IGFBP2 is not a driver of chemoresistance in these cells.

### IGFBP2-dependent effects of IGF1 on ERK and AKT activation

**Figure 4:**
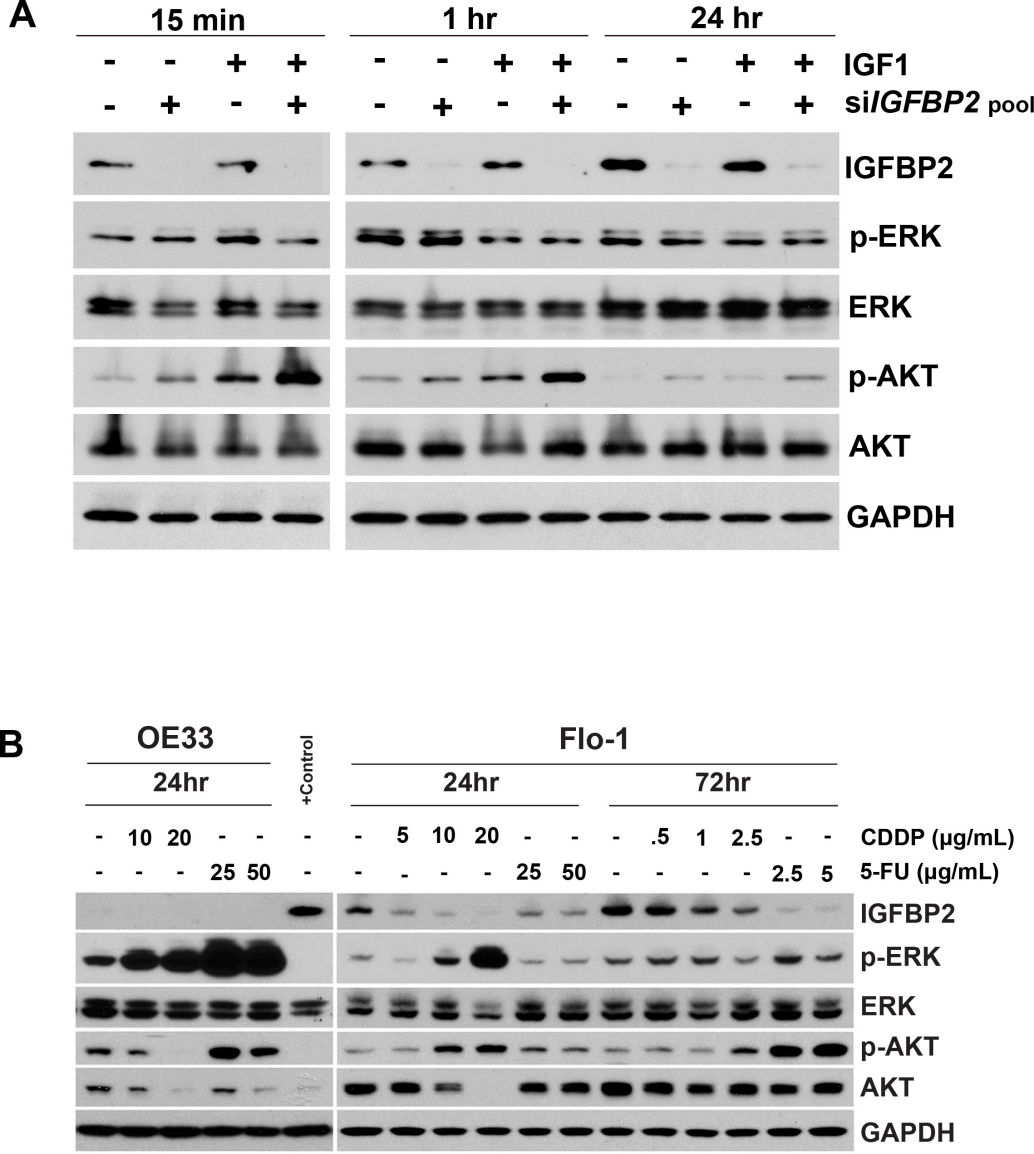

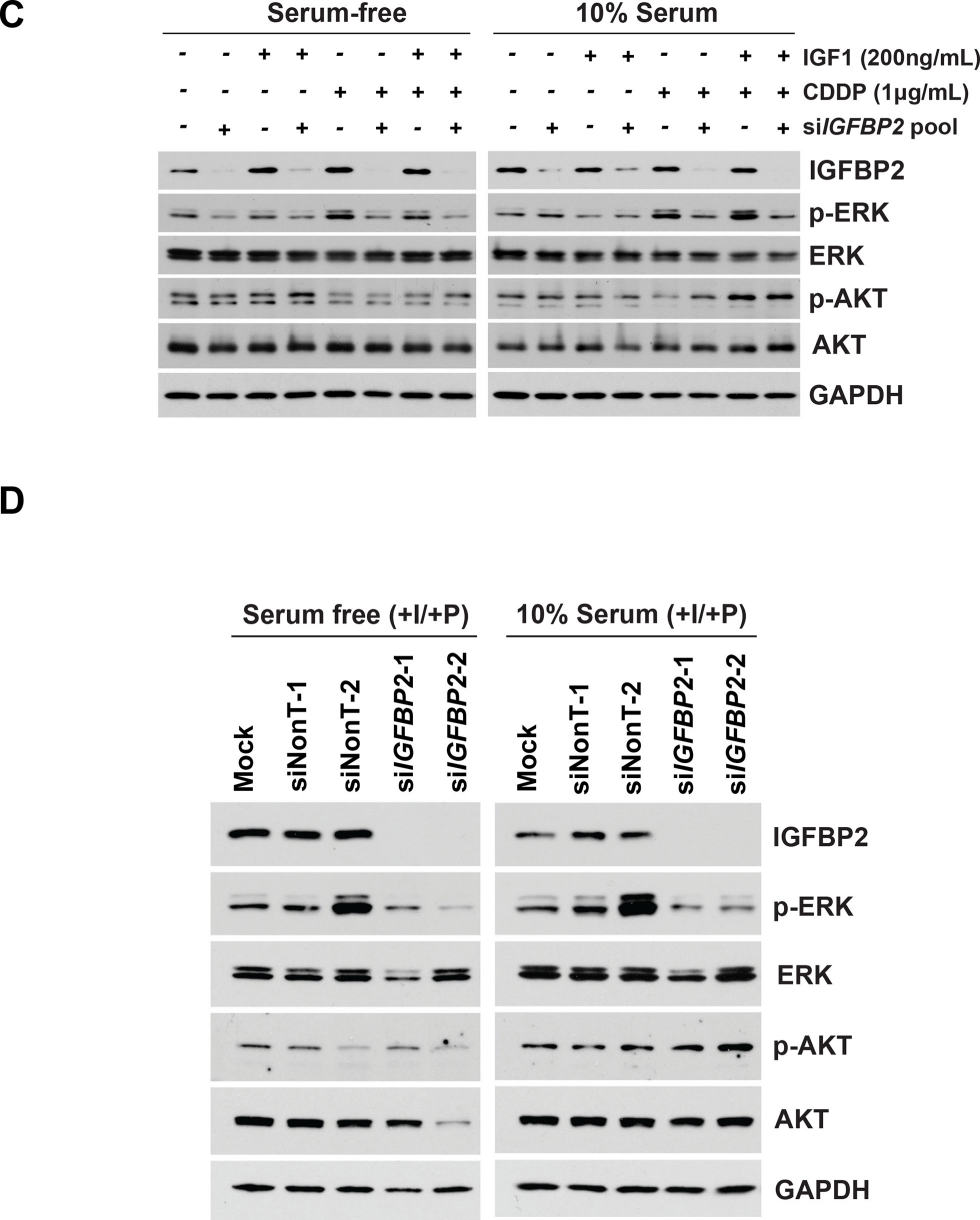
Effect of IGF1 and chemotherapy on expression of IGFBP2 and its downstream targets **A.** ON-TARGETplus *IGFBP2* siRNA SMARTpool- or siNon-Targeting control-treated Flo-1 cells were serum-starved for 24 hours followed by mock-treatment or treatment with 100 ng/mL IGF1 for 15 minutes, 1 hour, or 24 hours. **B.** Flo-1 and OE33 cells were mock-treated or treated with 5 μg/mL (16.7 μM) CDDP, 10 μg/mL (33.3 μM) CDDP, 20 μg/mL (66.7 μM) CDDP, 25 μg/mL (192.2 μM) 5-FU or 50 μg/mL (384.4 μM) 5-FU for 24 hours. Flo-1 cells were also mock-treated or treated with either 0.5 μg/mL (1.7 μM) CDDP, 1 μg/mL (3.3 μM) CDDP, 2.5 μg/mL (8.3uM) CDDP, 2.5 μg/mL (19.2 μM) 5-FU or 5 μg/mL (38.4 μM) 5-FU for 3 days. **C.** Following 24-hour treatment with ON-TARGETplus *IGFBP2* siRNA SMARTpool or siNon-Targeting control, Flo-1 cells were mock-pretreated or pretreated with 200 ng/mL IGF1 for 1 hour followed by mock-treatment or treatment with 1 μg/mL (3.3 μM) CDDP in serum-free or 10% serum DMEM for 3 days. **D.** Following 24-hour treatment with individual ON-TARGETplus *IGFBP2* siRNAs, siNon-Targeting controls or lipofectamine alone, Flo-1 cells were mock-pretreated or pretreated with 200 ng/mL IGF1 for 1 hour followed by mock-treatment or treatment with 1 μg/mL (3.3 μM) CDDP in serum-free or 10% serum DMEM for 3 days. Protein lysates were collected and Western blotting was performed to analyze changes in IGFBP2 expression and activated and total cellular levels of ERK and AKT. (I, 200 ng/mL IGF1; P, 1 μg/mL CDDP)

IGFBP2 has been shown to stimulate PI3K and MAPK activity in transformed cells by both IGF-dependent and IGF-independent mechanisms [19, 23, 32, 33, 35, 46]. To further explore the mechanism of IGFBP2-induced chemoresistance, we assessed whether inhibition of IGFBP2 could modulate IGF1-induced ERK and/or AKT activation (Figure 4A). Addition of recombinant IGF1 only transiently activated ERK and this activation at 15 minutes was attenuated by si*IGFBP2* treatment. IGF1 inhibited ERK activation at 1 hour in an IGFBP2-independent manner. In contrast, AKT was significantly activated by exogenous IGF1 or IGFBP2 knockdown at each measured time point as compared to the non-targeting siRNA control. Si*IGFBP2*-induced AKT activation was further enhanced by the addition of IGF1.

### Effects of chemotherapy on IGFBP2 and its effectors

To better understand the role of IGFBP2 in chemoresistance, we examined the effects of CDDP and 5-FU treatment alone on IGFBP2 and these critical signaling pathways. All three EAC cell lines used in this study were sensitive to CDDP. In Flo-1 cells, short-term treatment with higher doses and longer-term treatment with lower doses of CDDP or 5-FU both resulted in decreased levels of IGFBP2 (Figure 4B). OE33 cells were more sensitive to 3-day treatment with CDDP and also expressed much lower levels of IGFBP2 than Flo-1 cells (Figure 4B). Interestingly, in both Flo-1 and OE33 cell cultures, cells surviving repeated acute IC90 CDDP treatments had decreased expression of IGFBP2 (data not shown). OE19 cells were the most resistant to CDDP treatment. In contrast to Flo-1 and OE33 cells, *IGFBP2* mRNA expression levels in OE19 cells increased from lower baseline levels following repeated acute IC90 CDDP treatments (data not shown), while no difference in protein levels were detected when cells were treated with lower doses of CDDP for 3 days (Supplementary Figure S8). These data suggest that these 3 patient-derived EAC cell lines may utilize different mechanisms when developing resistance to chemotherapeutic agents such as CDDP.

Treatment of both Flo-1 and OE33 cells with high doses of CDDP for 24 hrs led to dose-dependent ERK activation, with much higher levels observed in OE33 cells. Treatment with 5-FU at higher doses dramatically induced ERK activation in OE33 but not Flo-1 cells. Acute CDDP treatment induced AKT activation in Flo-1 cells but inhibited activation in OE33 cells. Interestingly, total AKT expression was reduced in both CDDP-treated cell lines. Exposure of Flo-1 cells to low doses of either CDDP or 5-FU for 72 hours did not significantly alter ERK activation, but the highest dose of CDDP and both doses of 5-FU analyzed significantly increased AKT activation. These results suggest that EAC cell lines utilize different signaling pathways in their response to chemotherapy.

### ERK and AKT status during si*IGFBP2*-mediated chemosensitization

We then determined the impact of IGFBP2 inhibition on ERK or AKT signaling in IGF1- and/or CDDP-treated EAC cells (Figure 4C). IGFBP2-silenced Flo-1 cells were treated in the presence or absence of IGF1 and CDDP for 3 days. In serum-free conditions, ERK activation decreased following IGFBP2 knockdown. CDDP induced ERK activation in both serum-free and serum-replete conditions but this response was attenuated in cells treated with si*IGFBP2*. Only subtle changes in AKT activation were observed, suggesting that AKT activation may not be a sustained response to this combination treatment. Further examination of these signaling pathways using the individual siRNAs to *IGFBP2* in the SMARTpool confirmed that IGFBP2 knockdown reduced CDDP-induced ERK activation (Figure 4D). CDDP similarly induced ERK activation in OE19 cells (Supplementary Figure S8). IGFBP2 knockdown with the most effective individual si*IGFBP2* from the SMARTpool reduced ERK activation in the presence or absence of both IGF1 and CDDP. Effector differences among individual siRNAs in a SMARTpool have been previously reported [45] and may reflect differences in sequence-specific thermodynamics that translate into differences in effectiveness between oligos.

### Effect of small molecule inhibitors on si*IGFBP2*-mediated chemosensitization

**Figure 5:**
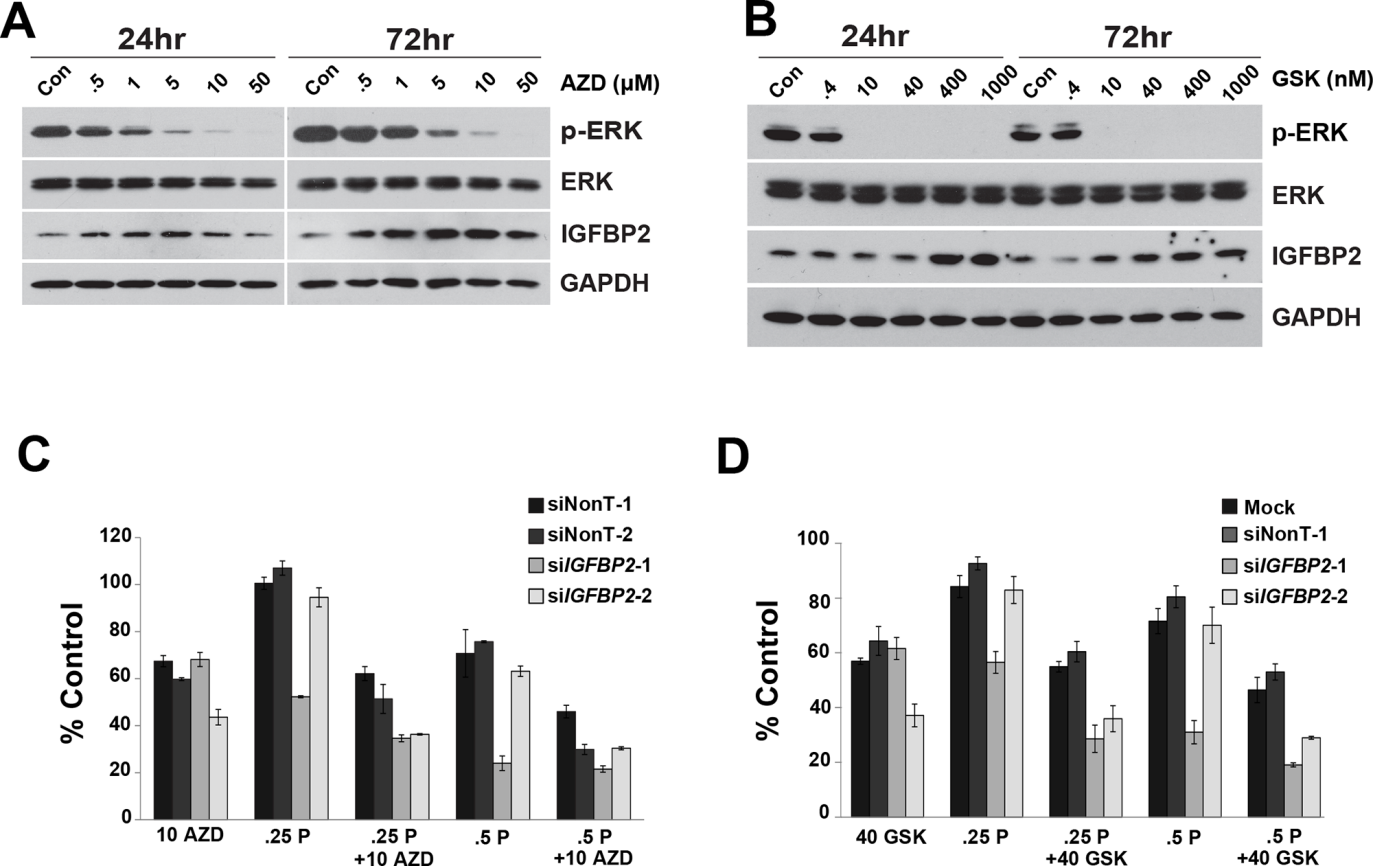
Effect of MEK1/2 Inhibitors on si*IGFBP2*-mediated chemosensitization Flo-1 cells were treated with increasing doses of either **A.** AZD6244 (AZD) or **B.** GSK1120212 (GSK) for 24 and 72 hours. Protein lysates were collected and Western blotting was performed to analyze ERK activation and IGFBP2 expression. Following 24-hour treatment with individual ON-TARGETplus *IGFBP2* siRNAs, siNon-Targeting controls or lipofectamine alone, Flo-1 cells were pretreated with 200 ng/mL IGF1 for 1 hour followed by mock-treatment or treatment with **C.** 10 μM AZD6244 or **D.** 40 nM GSK1120212 and, lastly, mock-treatment or treatment with 0.25 or 0.5 μg/mL (0.8 or 1.7 μM) CDDP in 10% serum DMEM for 3 days. WST analyses were performed to assess viability of treated cells. Columns and error bars are the mean ± SD of 3 or more wells in each experiment. (10 AZD, 10 μM AZD6244; 40 GSK, 40 nM GSK1120212; .25 P, 0.25 μg/mL CDDP; .5 P, 0.5 μg/mL CDDP)

In order to detect differences between single agents and combinations of agents in chemosensitization experiments, selected drug concentrations were optimized to capture additive/synergistic effects. Because CDDP treatment induced ERK phosphorylation in all 3 EAC cell lines, we assessed whether the MEK1/2 inhibitor, selumetinib, or the more potent MEK1/2 inhibitor, trametinib, would sensitize Flo-1 cells to CDDP following IGFBP2 knockdown. Doses that inhibited ERK1/2 activation effectively increased IGFBP2 expression (Figure 5A and 5B), reduced cell viability and enhanced CDDP sensitivity in both serum-free and serum-replete conditions. When combined with knockdown of IGFBP2, both selumetinib and trametinib increased sensitivity to CDDP in serum-replete (Figure 5C and 5D) but not in serum-free culture conditions (Supplementary Figure S9A and S9B).

**Figure 6:**
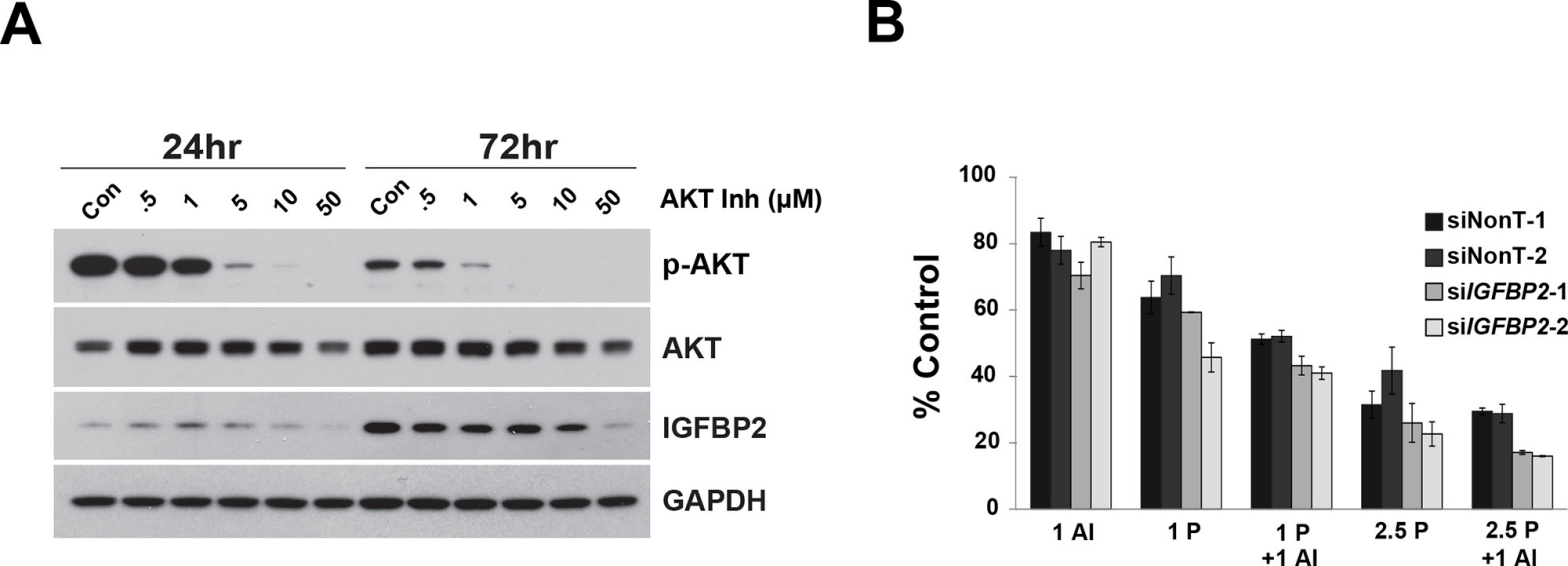
Effect of AKT Inhibitor VIII on si-*IGFBP2*-mediated chemosensitization **A.** Flo-1 cells were treated with increasing doses of AKT Inhibitor VIII (AI) for 24 and 72 hours. Protein lysates were collected and Western blotting was performed to analyze AKT activation and IGFBP2 expression. **B.** Following 24-hour treatment with individual ON-TARGETplus *IGFBP2* siRNAs, siNon-Targeting controls or lipofectamine alone, Flo-1 cells were pretreated with 200 ng/mL IGF1 for 1 hour followed by mock-treatment or treatment with 1 μM AKT Inhibitor VIII and, lastly, mock-treatment or treatment with 1 or 2.5 μg/mL (3.3 or 8.3 μM) CDDP in serum-free DMEM for 3 days. WST analyses were performed to assess viability of treated cells. Columns and error bars are the mean ± SD of 3 or more wells in each experiment. (1 AI, 1 μM AKT Inhibitor VIII; 1 P, 1 μg/mL CDDP; 2.5 P, 2.5 μg/mL CDDP)

Since knockdown of IGFBP2 induced AKT activation in Flo-1 cells, we hypothesized that inhibition of this pathway would further sensitize cells to CDDP. Doses of the potent, selective AKT Inhibitor VIII that completely abrogated AKT activation decreased IGFBP2 expression (Figure 6A) and dramatically reduced Flo-1 cell viability (Supplementary Figure S10). For combined inhibitor analyses, lower doses of inhibitor were necessary to capture additive/synergistic effects. In serum-free conditions, sensitization to CDDP was enhanced by addition of AKT Inhibitor VIII to si*IGFBP2*-treated cells (Figure 6B), suggesting that simultaneous modulation of AKT and IGFBP2 may be a useful treatment strategy. Although there was a slight dose-dependent decrease in cell viability following combined AKT Inhibitor VIII and si*IGFBP2* treatment, the addition of AKT Inhibitor VIII did not further enhance si*IGFBP2*-induced CDDP cytotoxicity in serum-replete conditions (Supplementary Figure S9C).

## DISCUSSION

Our clinical group and others have shown that the vast majority of esophageal cancers are resistant to existing chemotherapy options, which include treatment with platinum-based agents, taxanes and antimetabolites, with only 15–40% showing a pathological complete response [3–5] when combined with radiation therapy. As IGFBP2 has been shown to play pivotal roles in tumorigenesis, progression and chemoresistance in many organ systems and is a known modulator of two key activated signaling pathways in esophageal cancer [22–40], we examined its role in EAC progression and chemoresistance and its utility as a potential target for treatment of chemoresistant patients.

While *IGFBP2* expression varied across the spectrum of disease represented by the progression from dysplastic Barrett's esophagus to EAC, a subset of EAC samples expressed very high levels of *IGFBP2*. We explored whether IGFBP2 in such a subset of tumors might be associated with tumorigenesis or treatment resistance. In a cohort of patients who received chemotherapy following esophagectomy, *IGFBP2* expression was significantly higher in the resected EACs of patients with shorter disease-free intervals. Additionally, real-time PCR analysis showed that *IGFBP2* expression was higher among tumor specimens obtained from patients who had treatment-resistant EACs as compared with patients who had not yet received chemotherapy. The broad range of *IGFBP2* expression within the chemonaïve group, coupled with our finding that higher *IGFBP2* levels correlated with chemoresistance, suggests that a subset of these tumors may have exhibited resistance if treated. This idea is consistent with the observation that 20–30% of treated EACs show a high degree of resistance [3, 4]. It is also probable that mechanisms of treatment resistance in some of the tumors were independent of *IGFBP2* expression. As pretreatment biopsies were not available for the patients found to have treatment-resistant EACs, it is not clear whether higher *IGFBP2* levels in these patients were treatment-induced or inherent in patient tissues prior to treatment. We have shown, however, that CDDP and 5-FU treatments did not induce but rather inhibited IGFBP2 expression in Flo-1 and OE33 cell lines. Additionally, in a study of childhood acute lymphoblastic leukemia, elevated IGFBP2 serum levels remained high from diagnosis through intensive chemotherapy treatment for relapsed patients, while levels were significantly reduced in patients in remission [47], suggesting that high IGFBP2 level is prognostic of worse treatment response.

The three EAC cell lines Flo-1, OE19, and OE33 were evaluated in functional assays to investigate the role of IGFBP2 in EAC tumorigenesis and chemoresistance. Effective knockdown of IGFBP2 expression was obtained with *IGFBP2*-targeting siRNA in Flo-1 and OE19 cells while IGFBP2 re-expression was accomplished by stable transfection or exogenous supplementation in OE33 cells. Since serum deprivation is a common environmental condition in tumors and different pathways can be utilized based on serum status [48, 49], our studies were performed in both serum-free and serum-replete conditions. Additionally, as IGFBP2 is a known modulator of IGF signaling, we attempted to eliminate serum factors that could mask or interfere with our experimental manipulations. It has been previously reported that expression of downstream targets of the PI3K-AKT and EGFR-MAPK pathways increased following serum starvation in gliomas [50]. Serum starvation *in vitro* has been shown to reduce growth factor levels and has been reported to lead to a proliferation-quiescent status in normal cells while activating the cellular DNA damage response pathway in cancer cells [51]. Additionally, serum deprivation up-regulated invasion and the Na+/H+ exchanger (NHE), the principle pH-regulating mechanism in tumor cells, while serum replete conditions inhibited NHE activity and invasion of breast cancer cells in a PI3K-dependent manner [52]. Comparison of fifty fibroblast cultures derived from ten anatomical sites that were cultured in 10% serum versus 0.1% serum revealed a shared stereotypical fibroblast core serum response (CSR) that strongly correlated with a typical wound healing response [53]. The wound-like phenotype predicted increased risk of metastasis and death. Examination of this CSR in different tumor types found that although almost all prostate and hepatocellular carcinomas had the serum-induced signature, the CSR signature was evident in some breast, lung, and gastric carcinomas but absent in others, reemphasizing the importance of establishing response in the context of both serum-deplete and serum-replete conditions.

Silencing IGFBP2 in Flo-1 EAC cells resulted in significant sensitization to CDDP in serum-supplemented conditions, independent of the addition of IGF1 or IGF2, but was not as effective in serum-free conditions. This difference may be due to the fact that rapidly dividing cancer cells are more sensitive to chemotherapy and/or modulation but also that IGFBP2 may affect different pathways depending on the presence or absence of additional growth factors. Modulation of IGFBP2 expression was less effective in OE19 and OE33 cells, suggesting that IGFBP2 is not a driver of resistance in these *ERBB2 (HER2/neu)*-amplified [45] EAC cell lines.

Subtle differences in migration and invasion assays suggest that this gene may contribute to the metastatic potential of EAC cells. We also observed, however, that IGFBP2 knockdown alone and CDDP treatment itself induced the expression of several EMT-promoting genes in Flo-1 cells. This may warrant further exploration as this may confound the utility of this gene as a target to combat EAC chemoresistance.

IGFBP2 has been shown to reduce PTEN expression and enhance AKT activation in response to IGF1 through its heparin-binding domain [19]. It plays a key role in AKT pathway activation during glioma development and progression [32] and its knockdown has been shown to decrease IGF1-stimulated AKT activation in vascular smooth muscle cells [54] and acute leukemia cells [35]. In contrast to what has been previously reported, IGFBP2 knockdown activated AKT in Flo-1 EAC cells, and the effect was further enhanced by the addition of IGF1. We also observed an inverse correlation between IGFBP2 expression and AKT activation following acute high dose and 3-day low dose treatment with both CDDP and 5-FU. These observations and previously published data showing enhanced efficacy of receptor tyrosine kinase inhibitors and cytotoxic agents with AKT inhibition [55] prompted us to evaluate whether Akt inhibition might increase tumor cell response to CDDP. In the absence of serum but in the presence of IGF1, AKT inhibitor VIII further enhanced the effects of a 3-day treatment with CDDP and was even more effective following IGFBP2 knockdown, suggesting that the acute AKT activation following IGFBP2 knockdown may have been a compensatory survival mechanism. In fact, a compensatory activation of insulin receptor-AKT signaling has been previously reported with IGF-1R monoclonal antibody therapy in human tumor cells [56, 57]. In the presence of serum, however, inhibition of AKT phosphorylation did not improve response to chemotherapy over IGFBP2 knockdown alone, indicating that these mediators may be acting through the same signaling pathway or that other signaling pathways predominate in serum-replete conditions.

Modulation of IGFBP2 levels has been shown to affect ERK activation as well. Human recombinant IGFBP2 activated the ERK signaling pathway and stimulated proliferation in glioblastoma cells (through an integrin β1-dependent mechanism) [33] and ovarian cancer cells [58] and was shown to increase ERK as well as AKT activation in breast cancer cells [23]. Inhibition of the ERK pathway with PD98059 and U1026 abrogated IGFBP2-induced activation [33, 58]. While lentivirus-based shRNA silencing of IGFBP2 decreased activation of ERK and AKT in human leukemia cells [35], it resulted in the inability of p53 to inhibit IGF1-induced ERK phosphorylation in PC-3 cells [59]. We show that IGFBP2 knockdown also led to decreased ERK activation in Flo-1 and OE19 EAC cells, suggesting that ERK may be a downstream target of IGFBP2 in this tissue type as well. Interestingly, previous studies have shown that resistance to the IGF-1R inhibitor NVP-AEW541 may be driven by its inability to inhibit RAS/RAF/ERK activity in colon [60], biliary tract [61] and esophageal cancer [62]. In light of this and because CDDP treatment inhibited IGFBP2 expression but induced ERK activation in EAC cells, we tested whether MEK1/2 inhibitors could enhance si*IGFBP2*-induced CDDP cytotoxicity in Flo-1 cells. As opposed to AKT Inhibitor VIII, selumetinib and trametinib enhanced si*IGFBP2*-induced CDDP sensitization in serum-replete conditions but not in serum-free conditions. These data support the idea that activation of different signaling pathways can be dependent on serum status and that the effectiveness of targeted therapy may be dependent on the tumor microenvironment.

Despite promising data in preclinical models and a strong rationale for targeting the IGF signaling pathway in cancer, clinical efficacy of select IGF-1R inhibitors has been disappointing in multiple studies across many different tumor types [57]. Lack of targeted specificity, dependence on IGF1 levels and activation of compensatory pathways have all been implicated in the failure to improve current standard of care treatment. Of note, a phase I clinical trial using combination cixutumumab and selumetinib in advanced solid tumors showed preliminary evidence of clinical benefit [63]. IGFBP2 modulation is a promising alternative to IGF-1R inhibition due to its multi-faceted nature as a modulator of the IGF pathway, integrin signaling, cell-matrix interactions and transcription [16, 64]. Currently, a NCI-sponsored Phase I clinical trial is in progress to test the safety and immunogenicity of an IGFBP2-encoding DNA plasmid-based vaccine in patients with advanced stage or recurrent ovarian cancer, with the hopes of generating an IGFBP2-specific Th1 immune response (NCT01322802). Additional clinical trials are utilizing IGFBP2 as a potential immunotherapy target (NCT00821964) as well as a marker for cancer risk (NCT02450097), for colorectal cancer survival (NCT00503685) and for monitoring breast cancer treatment response (NCT01293032). Due to the established importance of both the IGF and integrin signaling pathways in esophageal cancer, IGFBP2 would be an ideal therapeutic target in the treatment of EAC. Our results linking increased IGFBP2 expression to chemoresistance in a subset of primary EACs and increased chemosensitization with its down-regulation in EAC cells makes it a strong candidate to combat resistant disease that so often claims the lives of patients.

In summary, oligonucleotide microarray and real-time PCR analyses implicated IGFBP2 as a potential mediator of chemoresistance in a subset of EACs. Further *in vitro* analyses of its role in EAC progression and chemoresistance showed that modulation of IGFBP2 expression affected proliferation, motility, and chemosensitization of EAC cells in a serum-dependent manner. Silencing of IGFBP2 affected both AKT and ERK activity and addition of targeted pharmacologic inhibitors of these pathways enhanced si*IGFBP2*-induced CDDP chemosensitization. Modulation of IGFBP2 in overexpressing EACs may be an effective approach to sensitizing resistant tumors to standard of care chemotherapy. Identification of such potential mechanisms of chemoresistance might have direct relevance for subjects currently being enrolled into therapeutic clinical trials, as these studies frequently include platinum-based therapy. While further *in vitro* studies and clinical validation will be needed, our findings suggest that characterization of IGFBP2 expression could inform treatment selection for subjects with esophageal adenocarcinoma, particularly for the majority of patients whose tumors do not demonstrate *HER2/neu* overexpression.

## MATERIALS AND METHODS

### Patients and tissues

Patients were reviewed by a multidisciplinary thoracic oncology tumor board and deemed eligible for chemoradiation and esophagectomy in terms of functional status, performance status (0–1) and tumor resectability. Written subject consent and approval of the Institutional Review Board were obtained to collect specimens from patients undergoing esophagectomy at the University of Michigan Medical Center (Ann Arbor MI). Specimens were transported to the laboratory in Dulbecco's modified Eagle's medium (DMEM; Invitrogen, Carlsbad CA) on ice. A portion of each sample was frozen in optimal cutting temperature (OCT) compound (Miles Inc., Elkhart IN) for cryostat sectioning. The remainder was frozen in liquid nitrogen and stored at −80°C. All tissue specimens were obtained from esophagectomies at the University of Michigan between 1991 and 2012 and were histologically classified as adenocarcinoma arising from either the gastroesophageal junction or the esophagus. Table 1 contains clinical information for all subjects whose tumors were analyzed in this study. Data were obtained primarily through our institutional cancer registry but supplemented with information from our prospective database and medical records.

### Cell lines and cell culture

Flo-1 was derived in our laboratory from a resected stage IIb EAC and has been described previously [65]. OE33 and OE19 were derived from stage IIa and III EAC patient tumors respectively, and are maintained by The European Collection of Cell Cultures (Sigma-Aldrich, Corp.; St Louis MO). Cell lines were grown in DMEM (Flo-1) or RPMI (OE33 and OE19) supplemented with 10% fetal bovine serum (FBS; Atlanta Biologicals, Norcross GA) and 1% penicillin/streptomycin/fungizone (Invitrogen) at 37°C in 5% carbon dioxide/95% air.

### Chemicals and antibodies

Cisplatin (cis-Diammineplatinum(II) dichloride; CDDP; Cat#: P4394) and 5-fluorouracil (5-FU; Cat#: F6627) were obtained from Sigma-Aldrich (St. Louis, MO). Recombinant Human IGFBP2 (Cat#: 674-B2), IGF1 (Cat#: 291-G1), and IGF2 (Cat#: 292-G2) were purchased from R&D Systems (Minneapolis, MN). Selumetinib (AZD6244; Cat#: S1008) and trametinib (GSK1120212; Cat#: S2673) were purchased from Selleck Chemicals (Houston, TX) and AKT Inhibitor VIII (Cat#: 124018) was purchased from EMD Millipore Corporation (San Diego, CA). Antibodies for phospho-ERK1/2 (Cat#: 4370; 1:2500 dilution), total ERK1/2 (Cat#: 9102; 1:3000 dilution), phospho-AKT (Cat#: 4060; 1:2000 dilution) and total AKT (Cat#: 4691; 1:3000 dilution) were obtained from Cell Signaling Technology (Beverly, Massachusetts), IGFBP2 antibody (Cat#: sc-6001; 1:500 dilution) was obtained from Santa Cruz Biotechnology (Dallas, TX) and GAPDH antibody (Cat#: MAB374; 1:10, 000 dilution) was obtained from Millipore (Billerica, MA). HRP-conjugated anti-goat secondary antibody (Cat#: HAF-109; 1:8000 dilution) was purchased from R&D Systems, HRP-conjugated anti-rabbit secondary antibody (Cat#: PI-1000; 1:20, 000 dilution) was purchased from Vector Laboratories (Burlingame, CA) and HRP-conjugated anti-mouse secondary antibody (Cat#: 1010-05; 1:20, 000 dilution) was purchased from Southern Biotech (Birmingham, AL). ON-TARGETplus Human IGFBP2 (Cat#: L-010-896-00-0005) and Non-targeting Control (Cat#s: D-001810-01 and D-001210-05) siRNAs were purchased from Dharmacon, Inc (Lafayette CO).

### Oligonucleotide microarray analysis

Total RNA was isolated from histologically normal esophageal squamous tissue, Barrett's esophageal tissue and EACs as previously described [66] using Trizol (Invitrogen) followed by RNeasy column purification (Qiagen, Valencia CA) per the manufacturers' instructions. cRNA was generated and hybridized to GeneChip HG-U133A oligonucleotide microarrays (Affymetrix, Santa Clara CA). Image analysis was performed by the University of Michigan DNA Microarray Core Facility. To normalize the microarray data, a summary statistic was calculated using the robust multichip average method [67] as implemented in the Affymetrix library of Bioconductor (version 1.3, www.bioconductor.org) which provides background adjustment, quantile normalization, and summarization. Normalized expression data was deposited to Gene Expression Omnibus (GEO), accession GSE37203 [68].

### Real-time PCR analysis

Total RNA from treated cells was isolated and column purified using the RNeasy Mini Kit (Qiagen) per the manufacturer's instructions. RNA was eluted from the spin column using RNase-free dH_2_O and reverse-transcribed using the High Capacity cDNA Reverse Transcription Kit (Cat#: 4368814) from Life Technologies (Waltham, MA) per the manufacturer's instructions. Real time PCR amplification using 20 ng total RNA, Power SYBR Green PCR Master Mix (Cat#: 4367659; Life Technologies), and 0.2 μM both forward and reverse primers (Supplementary Table S2) was performed on the Corbett Rotor-Gene 6000 (Qiagen) or the ABI PRISM 7900HT Sequence Detection System (Life Technologies) through the University of Michigan Sequencing Core. Annealing temperatures were optimized using the Cepheid SmartCycler (Cepheid, Sunnyvale, CA). Melt curves were monitored for nonspecific SYBR Green signals. Significant differences of relative quantification were determined using the 2^−ΔΔCt^ method [69] with normalization to GAPDH or β-actin.

### Immunohistochemistry and tissue microarray (TMA)

Tissue microarrays were constructed, as previously described [70], with formalin-fixed, paraffin-embedded tissues from 73 patients including 64 tumor, 8 lymph node metastases, 8 dysplastic Barrett's mucosa, and 11 nondysplastic Barrett's metaplasia samples. Multiple samples from representative areas of esophageal adenocarcinoma, metaplasia, or dysplasia were included for 33 patients. Normal esophagus was also included from 3 patients who had undergone esophagectomy for benign indications. Immunohistochemical staining was performed on the DAKO Autostainer (DAKO, Carpinteria, CA) using DAKO LSAB+ and diaminobenzadine (DAB) as the chromagen. Dewaxed and rehydrated sections of the TMA at 4 micron thickness were labeled with a 1:600 dilution of IGFBP2 antibody (Cat#: sc-6001; Santa Cruz Biotechnology). Microwave citric acid epitope retrieval was performed for 20 minutes. Slides were lightly counterstained with hematoxylin. Each sample was scored independently by two readers using a scale of 0 (no staining), 1+ (<10% staining), 2+ (10–50% staining), or 3+ (>50% staining).

### Protein extraction and immunoblot analysis

Total cellular protein was extracted in lysis buffer (150 mM NaCl; 20 mM Tris, pH 7.5; 1 mM EDTA; 1 mM EGTA; 2.5 mM Na_4_P_2_O_7_; 1 mM glycerol 2-phosphate disodium salt hydrate; 1 mM Na_3_VO_4_; 1% Triton X-100) supplemented with Protease Inhibitor Cocktail (20 μL/1 mL lysis buffer; Sigma-Aldrich) and centrifuged at 14,000 rpm, 4°C for 15 minutes. Twenty to 40 μg total cell lysate were boiled in 1x LDS Non-reducing Sample Buffer (Cat#: 84788; Life Technologies) supplemented with 5% 2-mercaptoethanol (Cat#:161-0710; Bio-Rad Laboratories, Hercules, CA), resolved on 4-20% Tris-glycine gels (Invitrogen), and transferred to Immobilon-P membranes (Millipore). Membranes were blocked with 5% Bovine Serum Albumin (Cat#: A7906, Sigma-Aldrich) or Blotting-grade Blocker (Cat#: 170-6404; Bio-Rad) for 1 hour at room temperature, incubated with primary antibody overnight at 4°C and subsequently with its respective secondary antibody at room temperature for 1 hour, and detected using Amersham ECL Prime Western Blotting Detection Reagent (Cat#: RPN2232; GE Healthcare Life Sciences, Piscataway, NJ).

### Construction of IGFBP2 stable cell line

An *IGFBP2* mammalian expression construct was created and stably tranfected into OE33 cells. *IGFBP2* was PCR-amplified from the cDNA of endogenously expressing Flo-1 cells using the GC-Rich PCR System (Roche, Indianapolis, IN) and primers containing EcoRI and BamHI restriction sites for directional cloning into the pEGFP-C1 vector (Clontech, Mountain View, CA). The pEGFP-*IGFBP2* or empty vector construct was transfected into OE33 cells using FuGENE 6 transfection reagent (Roche) per the manufacturer's instructions and selected with 1000 μg/mL Geneticin (Invitrogen). Colonies were ring-cloned, expanded and then maintained in growth media containing 200 μg/ml Geneticin.

### Proliferation/cytotoxicity assay

The cell proliferation reagent WST-1 (Roche) was used for spectrophotometric quantification of cell proliferation, viability, and chemosensitivity in accordance with the manufacturer's directions. Relative proliferation rates of EAC cell lines and chemosensitivity analyses in stably transfected OE33 cells were calculated as a percentage of the initial T_0_ reading within each cell line. T_0_ readings were measured 24 hours post-seeding and marked the initiation of respective treatments. Relative cell viability was calculated as OD_T_/OD_C_ × 100, where OD_T_ represented the absorbance of the treatment group and OD_C_ represented the absorbance of the control group.

### Wound healing assay

Cells were densely plated in triplicate and allowed to grow to 100% confluence. Following serum-starvation for 24 hours, cell monolayers were wounded with sterile p200 tips. Digital images of predetermined locations in each well were taken at both 4× optical magnification on an Olympus CK2 inverted microscope using a SPOT Idea 1.3 MP camera and analyzed with SPOT Basic software (Diagnostic Instruments, Sterling Heights, MI) immediately after wounding and subsequently until the wounds were closed. The percentage of initial wound width was determined by averaging measurements per well at each time point. These percentages were then averaged per group and compared across groups.

### Matrigel invasion assay

Ice-cold matrigel was diluted with coating buffer to a final concentration of 250 μg/mL. Diluted matrigel (100 μL) was added to each upper chamber of a 24-well transwell plate and allowed to gel at 37°C for 6 hours. Flo-1 cells that had been pretreated with si*IGFBP2* or Non-target siRNA control (Dharmacon) were trypsinized and resuspended in siRNA-containing serum-free DMEM to a final concentration of 40,000 cells in 500 μL and added to each transwell in duplicate. Pretreated cells were also added to uncoated transwells to serve as internal controls for each treatment group. Lower chambers were filled with 750 μL 10% FBS DMEM and plates were incubated at 37°C for 36 hours. Transwells were removed and stained with Diff-Quick solution. Noninvaded cells were scraped from the inside of the transwell with a cotton swab. Invaded cells were counted in 5 fields per transwell by light microscopy and percent invasion was calculated as I_C_/I_UNC_ × 100, where I_C_ represented total invaded cells counted per coated transwell and I_UNC_ represented total invaded cells counted per uncoated transwell for each treatment group.

## SUPPLEMENTARY FIGURES AND TABLES



## References

[1] Thrift AP, Whiteman DC (2012). The incidence of esophageal adenocarcinoma continues to rise: analysis of period and birth cohort effects on recent trends. Annals of oncology: official journal of the European Society for Medical Oncology/ESMO.

[2] Siegel R, Ma J, Zou Z, Jemal A (2014). Cancer statistics, 2014. CA: A Cancer Journal for Clinicians.

[3] Davies AR, Gossage JA, Zylstra J, Mattsson F, Lagergren J, Maisey N, Smyth EC, Cunningham D, Allum WH, Mason RC (2014). Tumor Stage After Neoadjuvant Chemotherapy Determines Survival After Surgery for Adenocarcinoma of the Esophagus and Esophagogastric Junction. J Clin Oncol.

[4] Orringer MB, Marshall B, Chang AC, Lee J, Pickens A, Lau CL (2007). Two thousand transhiatal esophagectomies: changing trends, lessons learned. Annals of Surgery.

[5] van Hagen P, Hulshof MC, van Lanschot JJ, Steyerberg EW, van Berge Henegouwen MI, Wijnhoven BP, Richel DJ, Nieuwenhuijzen GA, Hospers GA, Bonenkamp JJ, Cuesta MA, Blaisse RJ, Busch OR, ten Kate FJ, Creemers GJ, Punt CJ (2012). Preoperative chemoradiotherapy for esophageal or junctional cancer. The New England Journal of Medicine.

[6] Bahr C, Groner B (2005). The IGF-1 receptor and its contributions to metastatic tumor growth-novel approaches to the inhibition of IGF-1R function. Growth Factors.

[7] Chen SC, Chou CK, Wong FH, Chang CM, Hu CP (1991). Overexpression of epidermal growth factor and insulin-like growth factor-I receptors and autocrine stimulation in human esophageal carcinoma cells. Cancer Res.

[8] Iravani S, Zhang HQ, Yuan ZQ, Cheng JQ, Karl RC, Jove R, Coppola D (2003). Modification of insulin-like growth factor 1 receptor, c-Src, and Bcl-XL protein expression during the progression of Barrett's neoplasia. Human pathology.

[9] Liu YC, Leu CM, Wong FH, Fong WS, Chen SC, Chang C, Hu CP (2002). Autocrine stimulation by insulin-like growth factor I is involved in the growth, tumorigenicity and chemoresistance of human esophageal carcinoma cells. Journal of Biomedical Science.

[10] Kalinina T, Bockhorn M, Kaifi JT, Thieltges S, Gungor C, Effenberger KE, Strelow A, Reichelt U, Sauter G, Pantel K, Izbicki JR, Yekebas EF (2010). Insulin-like growth factor-1 receptor as a novel prognostic marker and its implication as a cotarget in the treatment of human adenocarcinoma of the esophagus. International Journal of Cancer Journal international du cancer.

[11] Sohda M, Kato H, Miyazaki T, Nakajima M, Fukuchi M, Manda R, Fukai Y, Masuda N, Kuwano H (2004). The role of insulin-like growth factor 1 and insulin-like growth factor binding protein 3 in human esophageal cancer. Anticancer Research.

[12] Doyle SL, Donohoe CL, Finn SP, Howard JM, Lithander FE, Reynolds JV, Pidgeon GP, Lysaght J (2012). IGF-1 and its receptor in esophageal cancer: association with adenocarcinoma and visceral obesity. The American Journal of Gastroenterology.

[13] Greer KB, Thompson CL, Brenner L, Bednarchik B, Dawson D, Willis J, Grady WM, Falk GW, Cooper GS, Li L, Chak A (2012). Association of insulin and insulin-like growth factors with Barrett's oesophagus. Gut.

[14] McElholm AR, McKnight AJ, Patterson CC, Johnston BT, Hardie LJ, Murray LJ, Finbar G (2010). A population-based study of IGF axis polymorphisms and the esophageal inflammation, metaplasia, adenocarcinoma sequence. Gastroenterology.

[15] Siahpush SH, Vaughan TL, Lampe JN, Freeman R, Lewis S, Odze RD, Blount PL, Ayub K, Rabinovitch PS, Reid BJ, Chen C (2007). Longitudinal study of insulin-like growth factor, insulin-like growth factor binding protein-3, and their polymorphisms: risk of neoplastic progression in Barrett's esophagus. Cancer Epidemiol Biomarkers Prev.

[16] Fukushima T, Kataoka H (2007). Roles of insulin-like growth factor binding protein-2 (IGFBP-2) in glioblastoma. Anticancer Research.

[17] Rajaram S, Baylink DJ, Mohan S (1997). Insulin-like growth factor-binding proteins in serum and other biological fluids: regulation and functions. Endocrine Reviews.

[18] Rosenzweig SA, Atreya HS (2010). Defining the pathway to insulin-like growth factor system targeting in cancer. Biochemical Pharmacology.

[19] Kawai M, Breggia AC, DeMambro VE, Shen X, Canalis E, Bouxsein ML, Beamer WG, Clemmons DR, Rosen CJ (2011). The heparin-binding domain of IGFBP-2 has insulin-like growth factor binding-independent biologic activity in the growing skeleton. The Journal of Biological Chemistry.

[20] Schutt BS, Langkamp M, Rauschnabel U, Ranke MB, Elmlinger MW (2004). Integrin-mediated action of insulin-like growth factor binding protein-2 in tumor cells. Journal of Molecular Endocrinology.

[21] Wang GK, Hu L, Fuller GN, Zhang W (2006). An interaction between insulin-like growth factor-binding protein 2 (IGFBP2) and integrin alpha5 is essential for IGFBP2-induced cell mobility. The Journal of Biological Chemistry.

[22] Sakamoto M, Kondo A, Kawasaki K, Goto T, Sakamoto H, Miyake K, Koyamatsu Y, Akiya T, Iwabuchi H, Muroya T, Ochiai K, Tanaka T, Kikuchi Y, Tenjin Y (2001). Analysis of gene expression profiles associated with cisplatin resistance in human ovarian cancer cell lines and tissues using cDNA microarray. Human Cell.

[23] Dokmanovic M, Shen Y, Bonacci TM, Hirsch DS, Wu WJ (2011). Trastuzumab regulates IGFBP-2 and IGFBP-3 to mediate growth inhibition: implications for the development of predictive biomarkers for trastuzumab resistance. Molecular Cancer Therapeutics.

[24] Juncker-Jensen A, Lykkesfeldt AE, Worm J, Ralfkiaer U, Espelund U, Jepsen JS (2006). Insulin-like growth factor binding protein 2 is a marker for antiestrogen resistant human breast cancer cell lines but is not a major growth regulator. Growth Hormone & IGF Research : official journal of the Growth Hormone Research Society and the International IGF Research Society.

[25] Sehgal P, Kumar N, Praveen Kumar VR, Patil S, Bhattacharya A, Vijaya Kumar M, Mukherjee G, Kondaiah P (2013). Regulation of protumorigenic pathways by insulin like growth factor binding protein2 and its association along with beta-catenin in breast cancer lymph node metastasis. Molecular Cancer.

[26] Fan CW, Chan CC, Chao CC, Fan HA, Sheu DL, Chan EC (2004). Expression patterns of cell cycle and apoptosis-related genes in a multidrug-resistant human colon carcinoma cell line. Scandinavian Journal of Gastroenterology.

[27] Hu Q, Huang L, Kuang X, Zhang H, Ling G, Chen X, Li K, Deng Z, Zhou J (2014). Is insulin-like growth factor binding protein 2 associated with metastasis in lung cancer?. Clinical & Experimental Metastasis.

[28] Lu H, Wang L, Gao W, Meng J, Dai B, Wu S, Minna J, Roth JA, Hofstetter WL, Swisher SG, Fang B (2013). IGFBP2/FAK pathway is causally associated with dasatinib resistance in non-small cell lung cancer cells. Molecular Cancer Therapeutics.

[29] Migita T, Narita T, Asaka R, Miyagi E, Nagano H, Nomura K, Matsuura M, Satoh Y, Okumura S, Nakagawa K, Seimiya H, Ishikawa Y (2010). Role of insulin-like growth factor binding protein 2 in lung adenocarcinoma: IGF-independent antiapoptotic effect via caspase-3. The American journal of Pathology.

[30] Degraff DJ, Aguiar AA, Sikes RA (2009). Disease evidence for IGFBP-2 as a key player in prostate cancer progression and development of osteosclerotic lesions. American journal of Translational Research.

[31] Uzoh CC, Holly JM, Biernacka KM, Persad RA, Bahl A, Gillatt D, Perks CM (2011). Insulin-like growth factor-binding protein-2 promotes prostate cancer cell growth via IGF-dependent or -independent mechanisms and reduces the efficacy of docetaxel. British Journal of Cancer.

[32] Dunlap SM, Celestino J, Wang H, Jiang R, Holland EC, Fuller GN, Zhang W (2007). Insulin-like growth factor binding protein 2 promotes glioma development and progression. Proceedings of the National Academy of Sciences of the United States of America.

[33] Han S, Li Z, Master LM, Master ZW, Wu A (2014). Exogenous IGFBP-2 promotes proliferation, invasion, and chemoresistance to temozolomide in glioma cells via the integrin beta1-ERK pathway. British Journal of Cancer.

[34] Wang H, Wang H, Shen W, Huang H, Hu L, Ramdas L, Zhou YH, Liao WS, Fuller GN, Zhang W (2003). Insulin-like growth factor binding protein 2 enhances glioblastoma invasion by activating invasion-enhancing genes. Cancer Res.

[35] Chen X, Zheng J, Zou Y, Song C, Hu X, Zhang CC (2013). IGF binding protein 2 is a cell-autonomous factor supporting survival and migration of acute leukemia cells. Journal of Hematology and Oncology.

[36] Kuhnl A, Kaiser M, Neumann M, Fransecky L, Heesch S, Radmacher M, Marcucci G, Bloomfield CD, Hofmann WK, Thiel E, Baldus CD (2011). High expression of IGFBP2 is associated with chemoresistance in adult acute myeloid leukemia. Leukemia Research.

[37] Vorwerk P, Mohnike K, Wex H, Rohl FW, Zimmermann M, Blum WF, Mittler U (2005). Insulin-like growth factor binding protein-2 at diagnosis of childhood acute lymphoblastic leukemia and the prediction of relapse risk. The Journal of Clinical Endocrinology and Metabolism.

[38] Lee EJ, Mircean C, Shmulevich I, Wang H, Liu J, Niemisto A, Kavanagh JJ, Lee JH, Zhang W (2005). Insulin-like growth factor binding protein 2 promotes ovarian cancer cell invasion. Molecular Cancer.

[39] Becher OJ, Peterson KM, Khatua S, Santi MR, MacDonald TJ (2008). IGFBP2 is overexpressed by pediatric malignant astrocytomas and induces the repair enzyme DNA-PK. Journal of Child Neurology.

[40] Rajeshkumar NV, Tan AC, De Oliveira E, Womack C, Wombwell H, Morgan S, Warren MV, Walker J, Green TP, Jimeno A, Messersmith WA, Hidalgo M (2009). Antitumor effects and biomarkers of activity of AZD0530, a Src inhibitor, in pancreatic cancer. Clin Cancer Res.

[41] Christiansen JJ, Rajasekaran AK (2006). Reassessing epithelial to mesenchymal transition as a prerequisite for carcinoma invasion and metastasis. Cancer Res.

[42] Kang Y, Massague J (2004). Epithelial-mesenchymal transitions: twist in development and metastasis. Cell.

[43] Gamelin EC, Danquechin-Dorval EM, Dumesnil YF, Maillart PJ, Goudier MJ, Burtin PC, Delva RG, Lortholary AH, Gesta PH, Larra FG (1996). Relationship between 5-fluorouracil (5-FU) dose intensity and therapeutic response in patients with advanced colorectal cancer receiving infusional therapy containing 5-FU. Cancer.

[44] Katano K, Tsujitani S, Oka S, Saito H, Gomyo Y, Kondo A, Ikeguchi M, Maeta M, Kaibara N (2000). Pharmacokinetics of hypotonic cisplatin chemotherapy administered into the peritoneal and the pleural cavities in experimental model. Anticancer Research.

[45] Wetterskog D, Shiu KK, Chong I, Meijer T, Mackay A, Lambros M, Cunningham D, Reis-Filho JS, Lord CJ, Ashworth A (2014). Identification of novel determinants of resistance to lapatinib in ERBB2-amplified cancers. Oncogene.

[46] Moore MG, Wetterau LA, Francis MJ, Peehl DM, Cohen P (2003). Novel stimulatory role for insulin-like growth factor binding protein-2 in prostate cancer cells. International Journal of Cancer journal international du cancer.

[47] Kitszel A, Krawczuk-Rybak M (2007). Are elevated serum levels of IGFBP-2 after intensive chemotherapy of childhood acute lymphoblastic leukemia a risk factor of relapse?. Advances in Medical Sciences.

[48] Brown JM (2007). Tumor hypoxia in cancer therapy. Methods in enzymology.

[49] Shi L, Wang S, Zangari M, Xu H, Cao TM, Xu C, Wu Y, Xiao F, Liu Y, Yang Y, Salama M, Li G, Tricot G, Zhan F (2010). Over-expression of CKS1B activates both MEK/ERK and JAK/STAT3 signaling pathways and promotes myeloma cell drug-resistance. Oncotarget.

[50] Levin VA, Panchabhai SC, Shen L, Kornblau SM, Qiu Y, Baggerly KA (2010). Different changes in protein and phosphoprotein levels result from serum starvation of high-grade glioma and adenocarcinoma cell lines. Journal of Proteome Research.

[51] Shi Y, Felley-Bosco E, Marti TM, Orlowski K, Pruschy M, Stahel RA (2012). Starvation-induced activation of ATM/Chk2/p53 signaling sensitizes cancer cells to cisplatin. BMC Cancer.

[52] Reshkin SJ, Bellizzi A, Albarani V, Guerra L, Tommasino M, Paradiso A, Casavola V (2000). Phosphoinositide 3-kinase is involved in the tumor-specific activation of human breast cancer cell Na(+)/H(+) exchange, motility, and invasion induced by serum deprivation. The Journal of Biological Chemistry.

[53] Chang HY, Sneddon JB, Alizadeh AA, Sood R, West RB, Montgomery K, Chi JT, van de Rijn M, Botstein D, Brown PO (2004). Gene expression signature of fibroblast serum response predicts human cancer progression: similarities between tumors and wounds. PLoS Biology.

[54] Shen X, Xi G, Maile LA, Wai C, Rosen CJ, Clemmons DR (2012). Insulin-like growth factor (IGF) binding protein 2 functions coordinately with receptor protein tyrosine phosphatase beta and the IGF-I receptor to regulate IGF-I-stimulated signaling. Molecular and Cellular Biology.

[55] Hirai H, Sootome H, Nakatsuru Y, Miyama K, Taguchi S, Tsujioka K, Ueno Y, Hatch H, Majumder PK, Pan BS, Kotani H (2010). MK-2206, an allosteric Akt inhibitor, enhances antitumor efficacy by standard chemotherapeutic agents or molecular targeted drugs *in vitro* and *in vivo*. Molecular Cancer Therapeutics.

[56] Buck E, Gokhale PC, Koujak S, Brown E, Eyzaguirre A, Tao N, Rosenfeld-Franklin M, Lerner L, Chiu MI, Wild R, Epstein D, Pachter JA, Miglarese MR (2010). Compensatory insulin receptor (IR) activation on inhibition of insulin-like growth factor-1 receptor (IGF-1R): rationale for cotargeting IGF-1R and IR in cancer. Molecular Cancer Therapeutics.

[57] Pillai RN, Ramalingam SS (2013). Inhibition of insulin-like growth factor receptor: end of a targeted therapy?. Translational Lung Cancer Research.

[58] Chakrabarty S, Kondratick L (2006). Insulin-like growth factor binding protein-2 stimulates proliferation and activates multiple cascades of the mitogen-activated protein kinase pathways in NIH-OVCAR3 human epithelial ovarian cancer cells. Cancer Biology & Therapy.

[59] Grimberg A, Coleman CM, Shi Z, Burns TF, MacLachlan TK, Wang W, El-Deiry WS (2006). Insulin-like growth factor factor binding protein-2 is a novel mediator of p53 inhibition of insulin-like growth factor signaling. Cancer Biology & Therapy.

[60] Cunningham MP, Thomas H, Marks C, Green M, Fan Z, Modjtahedi H (2008). Co-targeting the EGFR and IGF-IR with anti-EGFR monoclonal antibody ICR62 and the IGF-IR tyrosine kinase inhibitor NVP-AEW541 in colorectal cancer cells. International journal of Oncology.

[61] Wolf S, Lorenz J, Mossner J, Wiedmann M (2010). Treatment of biliary tract cancer with NVP-AEW541: mechanisms of action and resistance. World Journal of Gastroenterology: WJG.

[62] Bao XH, Takaoka M, Hao HF, Wang ZG, Fukazawa T, Yamatsuji T, Sakurama K, Sun DS, Nagasaka T, Fujiwara T, Naomoto Y (2012). Esophageal cancer exhibits resistance to a novel IGF-1R inhibitor NVP-AEW541 with maintained RAS-MAPK activity. Anticancer Research.

[63] Wilky BA, Rudek MA, Ahmed S, Laheru DA, Cosgrove D, Donehower RC, Nelkin B, Ball D, Doyle LA, Chen H, Ye X, Bigley G, Womack C, Azad NS (2015). A phase I trial of vertical inhibition of IGF signalling using cixutumumab, an anti-IGF-1R antibody, and selumetinib, an MEK 1/2 inhibitor, in advanced solid tumours. British journal of Cancer.

[64] Hoeflich A, Reisinger R, Lahm H, Kiess W, Blum WF, Kolb HJ, Weber MM, Wolf E (2001). Insulin-like growth factor-binding protein 2 in tumorigenesis: protector or promoter?. Cancer Res.

[65] Lin L, Bass AJ, Lockwood WW, Wang Z, Silvers AL, Thomas DG, Chang AC, Lin J, Orringer MB, Li W, Glover TW, Giordano TJ, Lam WL, Meyerson M, Beer DG (2012). Activation of GATA binding protein 6 (GATA6) sustains oncogenic lineage-survival in esophageal adenocarcinoma. Proceedings of the National Academy of Sciences of the United States of America.

[66] Lin J, Raoof DA, Wang Z, Lin MY, Thomas DG, Greenson JK, Giordano TJ, Orringer MB, Chang AC, Beer DG, Lin L (2006). Expression and effect of inhibition of the ubiquitin-conjugating enzyme E2C on esophageal adenocarcinoma. Neoplasia.

[67] Irizarry RA, Hobbs B, Collin F, Beazer-Barclay YD, Antonellis KJ, Scherf U, Speed TP (2003). Exploration, normalization, and summaries of high density oligonucleotide array probe level data. Biostatistics.

[68] Silvers AL, Lin L, Bass AJ, Chen G, Wang Z, Thomas DG, Lin J, Giordano TJ, Orringer MB, Beer DG, Chang AC (2010). Decreased selenium-binding protein 1 in esophageal adenocarcinoma results from posttranscriptional and epigenetic regulation and affects chemosensitivity. Clin Cancer Res.

[69] Livak KJ, Schmittgen TD (2001). Analysis of relative gene expression data using real-time quantitative PCR and the 2(−Delta Delta C(T)) Method. Methods.

[70] Kononen J, Bubendorf L, Kallioniemi A, Barlund M, Schraml P, Leighton S, Torhorst J, Mihatsch MJ, Sauter G, Kallioniemi OP (1998). Tissue microarrays for high-throughput molecular profiling of tumor specimens. Nat Med.

