# Supplementary material for: IGFBP2 modulates the chemoresistant phenotype in esophageal adenocarcinoma
Source: Oncotarget. 2015 Jul 17;6(28):25897–916. doi: 10.18632/oncotarget.4532 (PMC4694874; doi:10.18632/oncotarget.4532)
Supplement: Supplementary file 1 [file oncotarget-06-25897-s001.pdf]

SUPPLEMENTARY FIGURES AND TABLES

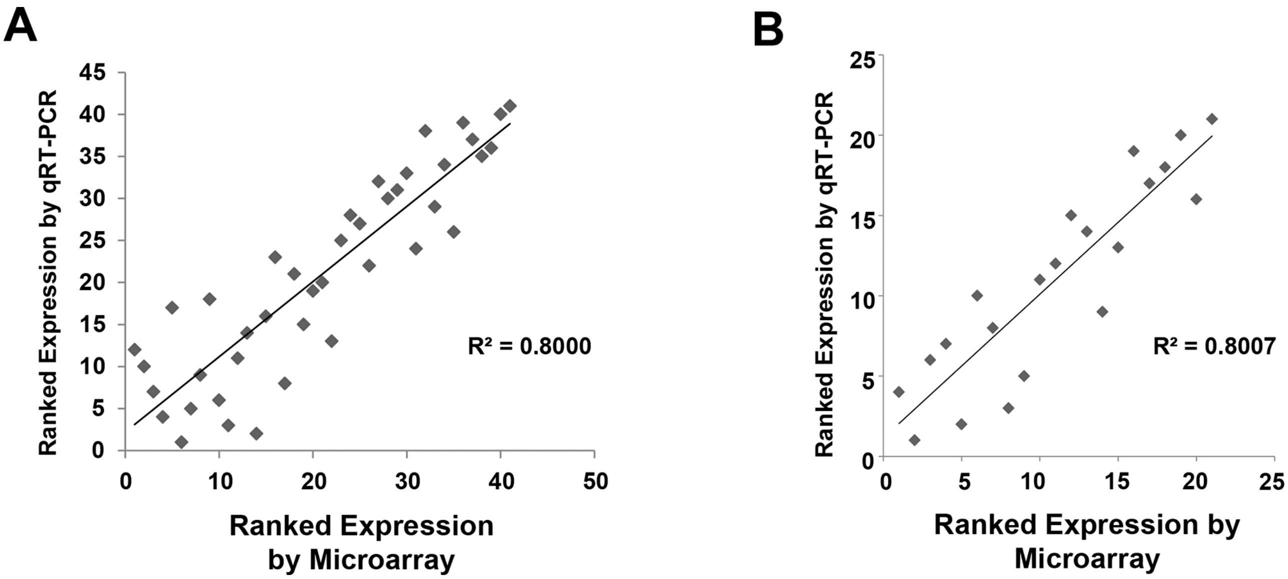

Supplementary Figure S1: Correlation of *IGFBP2* expression by Affymetrix HG-U133A oligonucleotide microarray (K_202718_at) and real-time PCR in esophageal tissues and EACs. mRNAs from **A.** the progression series of human esophageal tissues ranging from Barrett's metaplasia, low-grade dysplasia, high-grade dysplasia to adenocarcinoma and **B.** the cohort of disease-free versus recurrent disease chemonaïve EACs were reverse transcribed into cDNA followed by real-time PCR amplification using primers to *IGFBP2* and *GAPDH*. Relative quantification was determined using the $2^{-\Delta\Delta Ct}$ method, and expression values were calculated relative to the Barrett's metaplasia mean in the progression cohort and the overall tumor mean in the chemonaïve cohort. These values were then ranked within each analysis and correlated between analyses.

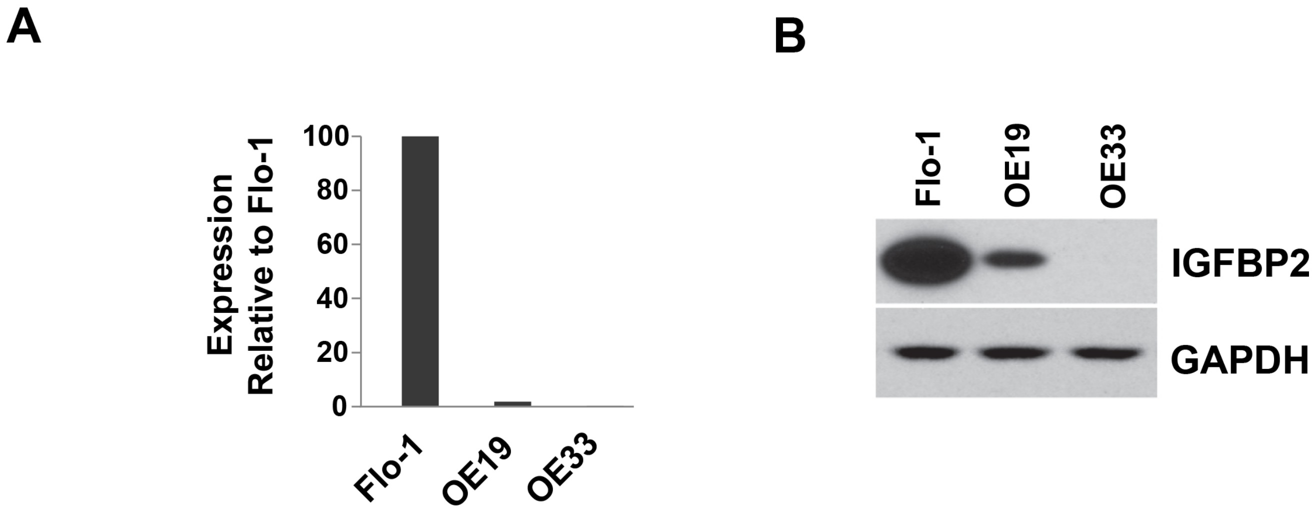

Supplementary Figure S2: IGFBP2 expression in EAC cell lines. Endogenous IGFBP2 expression in EAC cell lines examined by **A.** qRT-PCR and **B.** Western blotting.

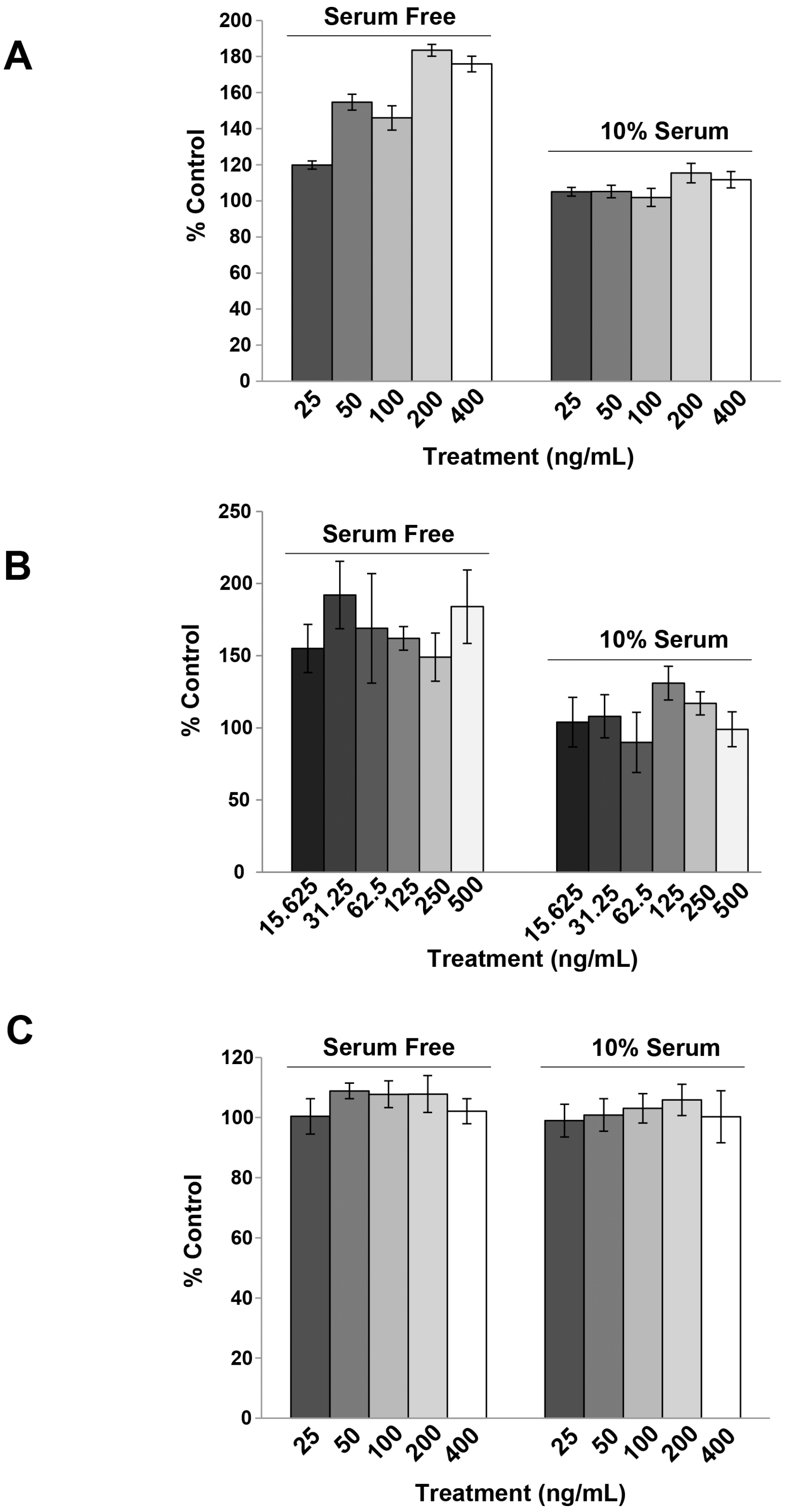

Supplementary Figure S3: Effect of exogenous IGF1 on EAC cell lines. WST analysis of EAC cells that were treated 24 hours post-seeding with increasing doses of IGF1 in serum-free or 10% serum media for 3 days. **A.** Flo-1 cells **B.** OE33 cells **C.** OE19 cells. Columns and error bars are the mean ± SD of 3 or more wells in each experiment.

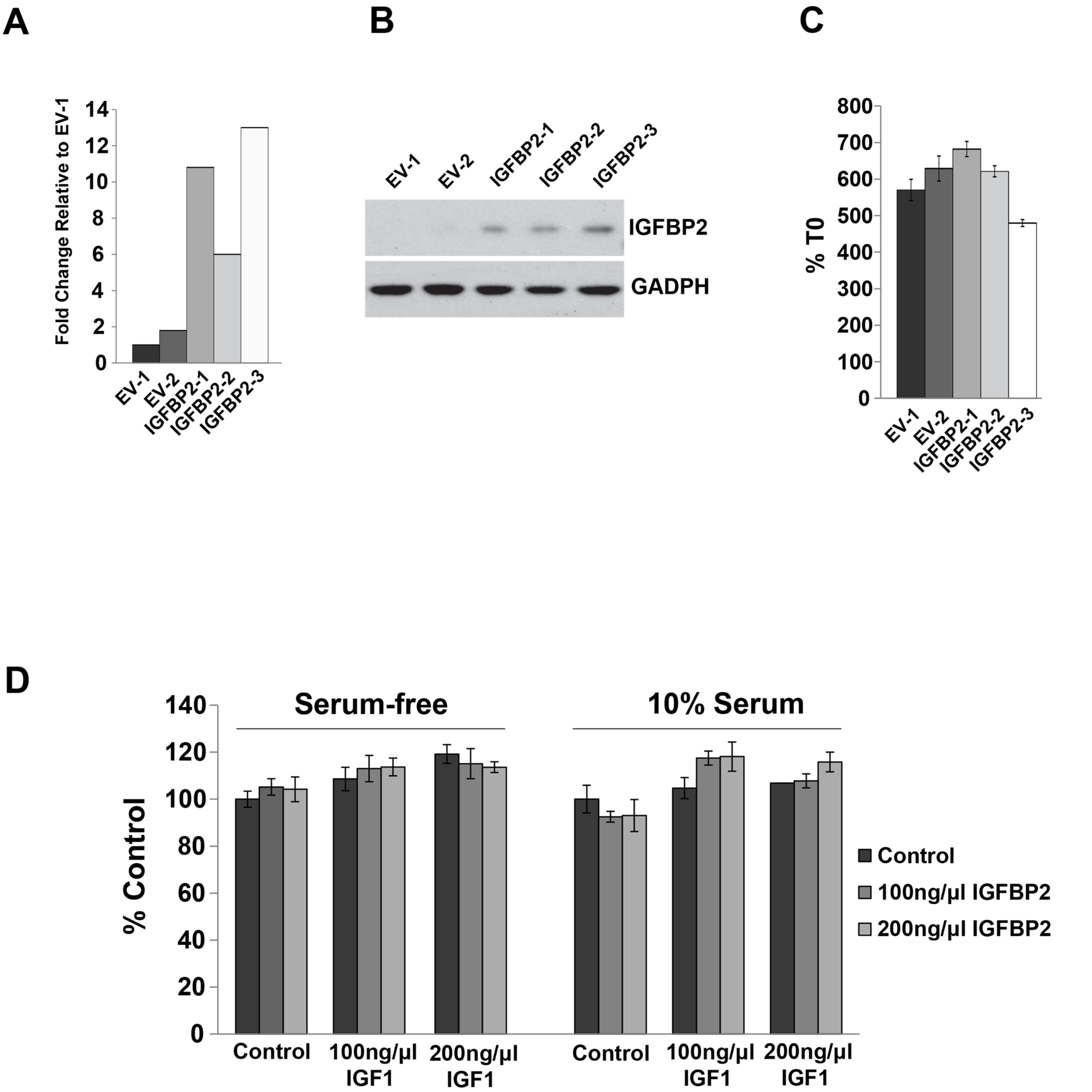

Supplementary Figure S4: Proliferative effect of IGFBP2 in OE33 EAC cells. A. Real-time PCR and **B.** western blot analyses of IGFBP2 expression in OE33 cells which had been stably-transfected with either the pEGFP-C1 empty vector or the pEGFP-C1-IGFBP2 expression construct and clonally selected using 1000 µg/mL Geneticin. **C.** WST analysis of the proliferative capacity of OE33 stable transfectants. WST readings were taken 24 (T0) and 96 (T72) hours post-seeding and clonal proliferative rates were calculated as the percentage of T0. Columns and error bars are the mean ± SD of 3 or more wells in each experiment. **D.** WST analysis of OE33 cells mock-pretreated or pretreated with human recombinant IGFBP2 followed by mock treatment or treatment with human recombinant IGF1 for 3 days. Columns and error bars are the mean ± SD of 3 or more wells in each experiment.

A

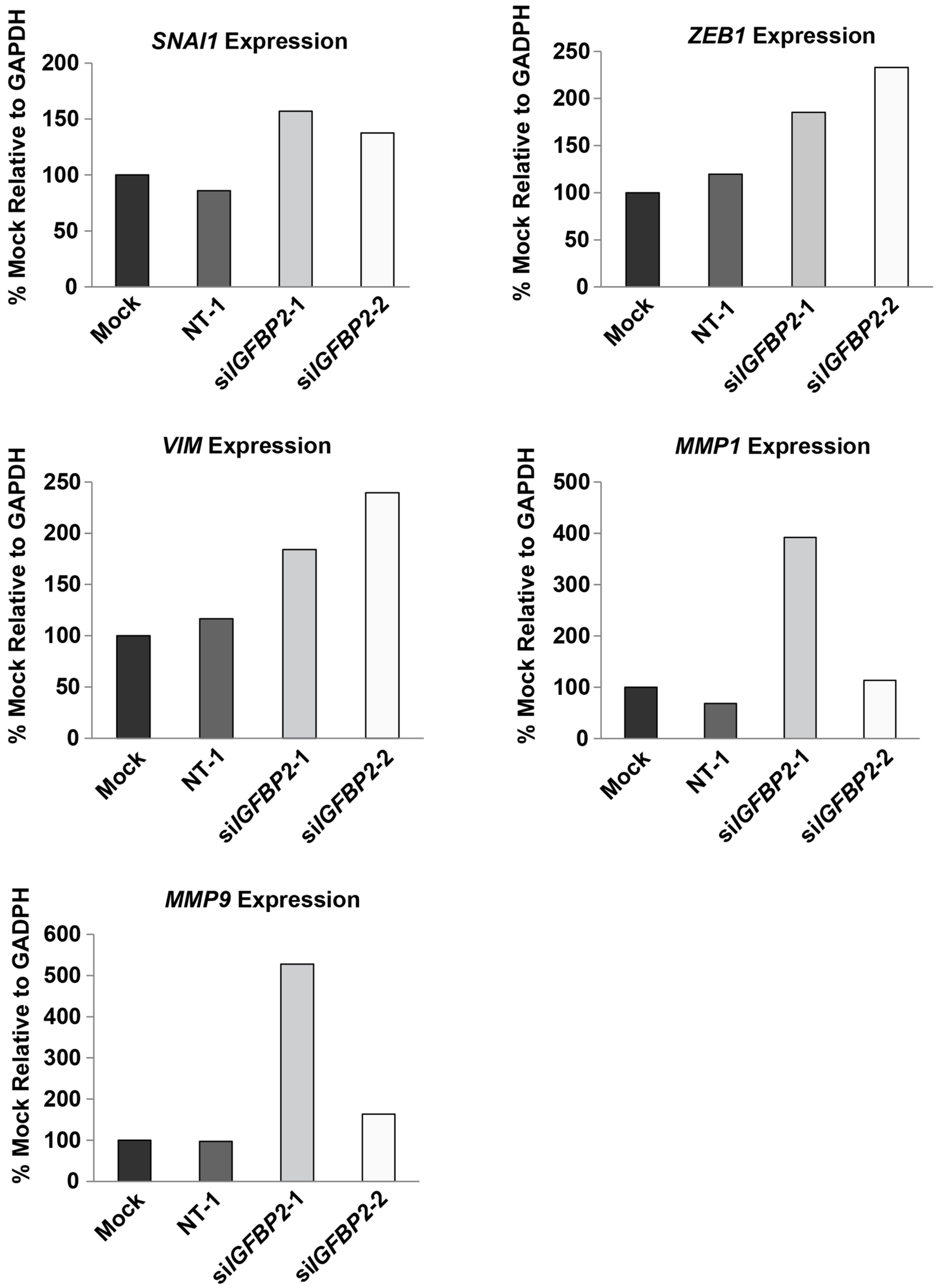

Supplementary Figure S5: Correlation of *IGFBP2* and EMT-related gene expression. Real-time PCR analysis of **A.** *SNAI1, ZEB1, MMP9, MMP1* and *VIM* expression in Flo-1 cells treated with individual ON-TARGETplus *IGFBP2* siRNAs, an siNon-targeting control, or lipofectamine alone; (*Continued*)

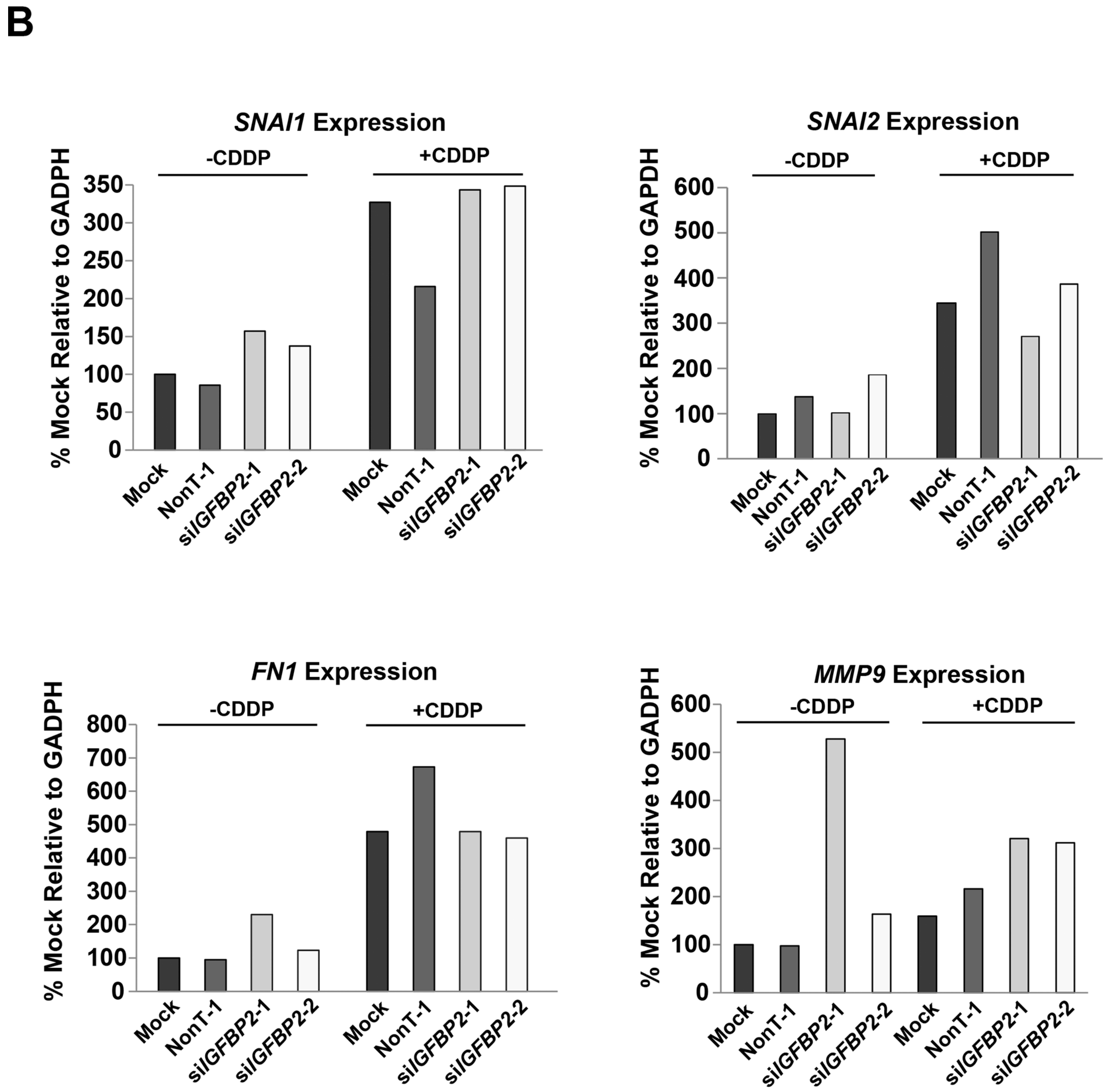

Supplementary Figure S5: (*Continued*) Correlation of *IGFBP2* and EMT-related gene expression. B. *SNAI1, SNAI2, FN1, CDH1*, and *VIM* expression in Flo-1 cells treated with individual ON-TARGETplus *IGFBP2* siRNAs, an siNon-targeting control, or lipofectamine alone in the presence or absence of 1 μg/mL (3.3 μM) CDDP;

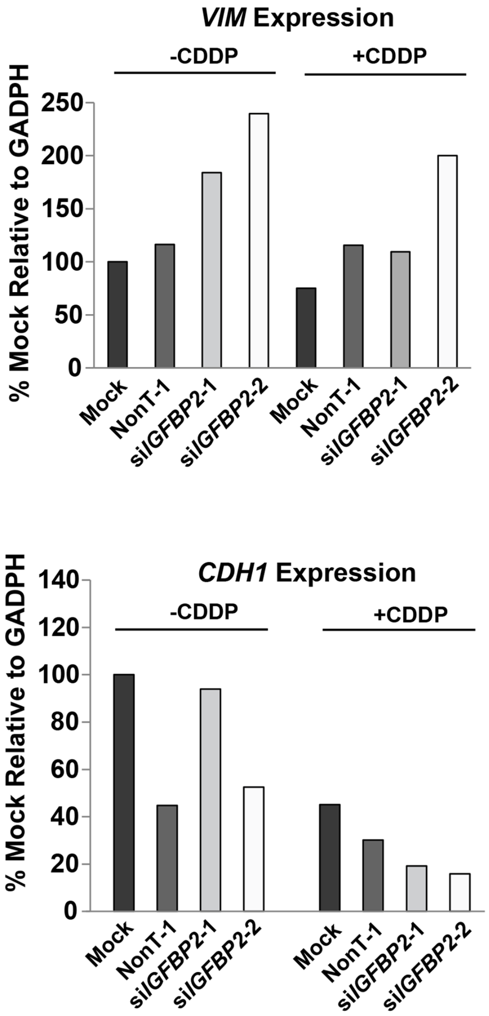

C

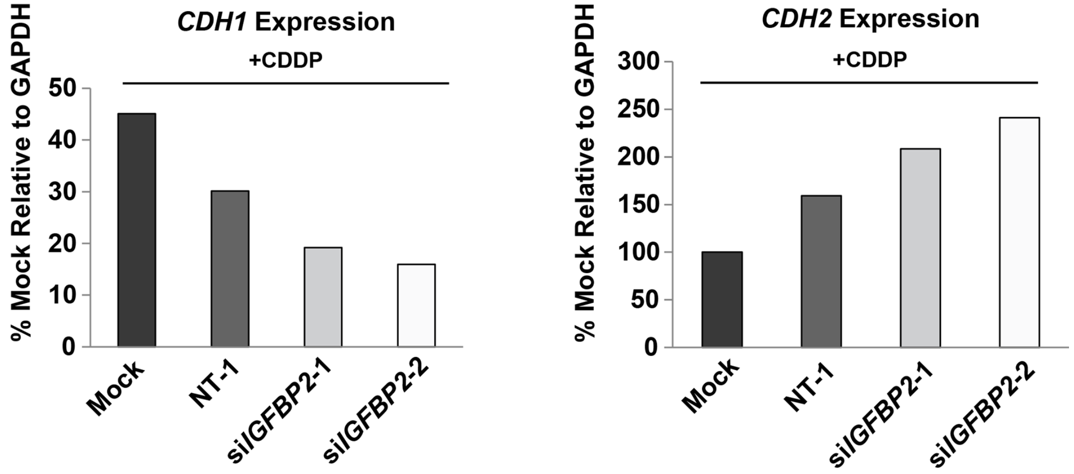

Supplementary Figure S5: (*Continued*) Correlation of *IGFBP2* and EMT-related gene expression. C. *CDH1* and *CDH2* expression in Flo-1 cells treated with individual ON-TARGETplus *IGFBP2* siRNAs, an siNon-targeting control, or lipofectamine alone in the presence of 1 µg/mL (3.3 µM) CDDP.

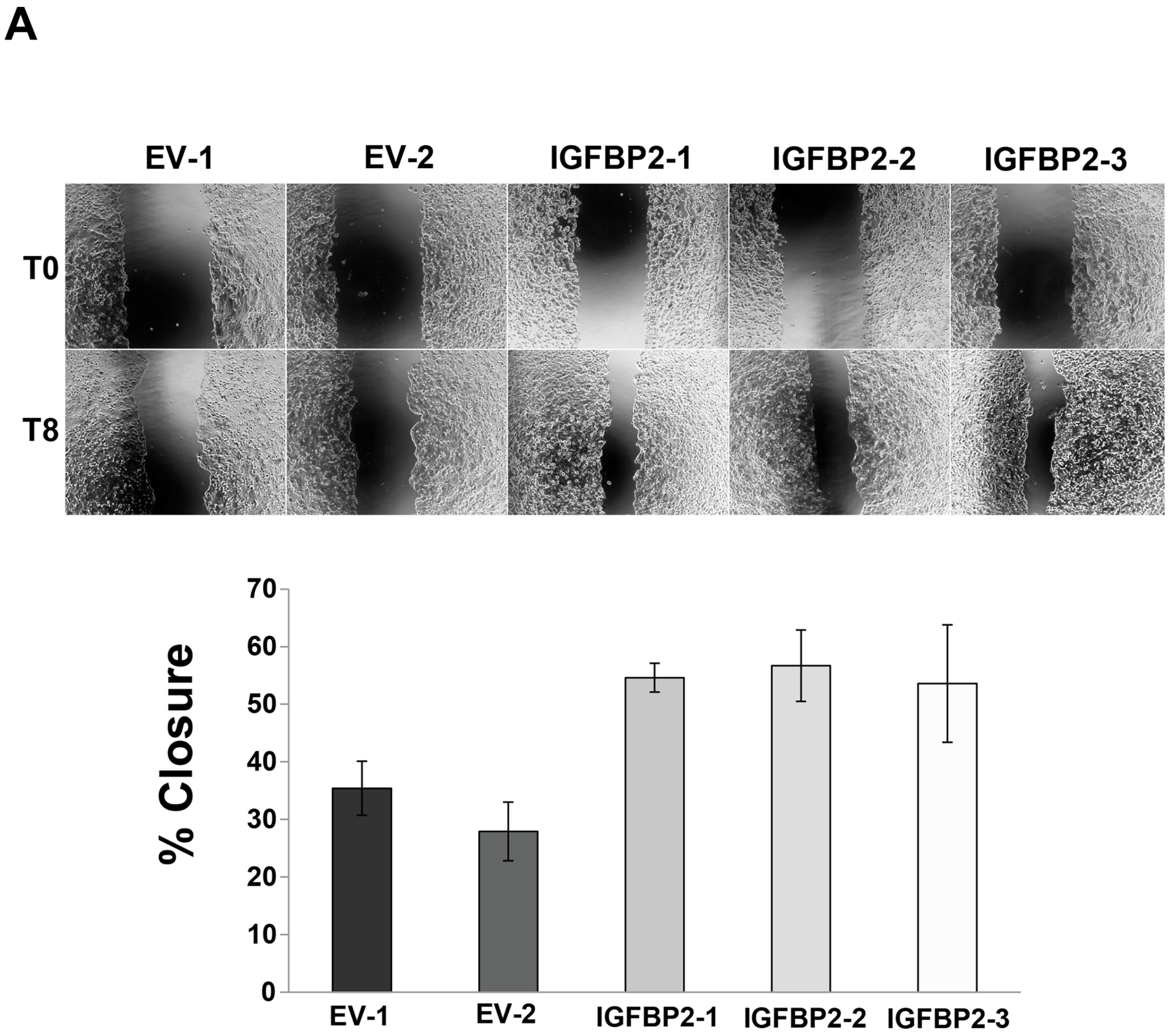

Supplementary Figure S6: IGFBP2-mediated migratory and invasive capacity of EAC cells. Densely plated **A.** OE33-empty vector and -IGFBP2 stable transfectants or **B.** (*Continued*)

B

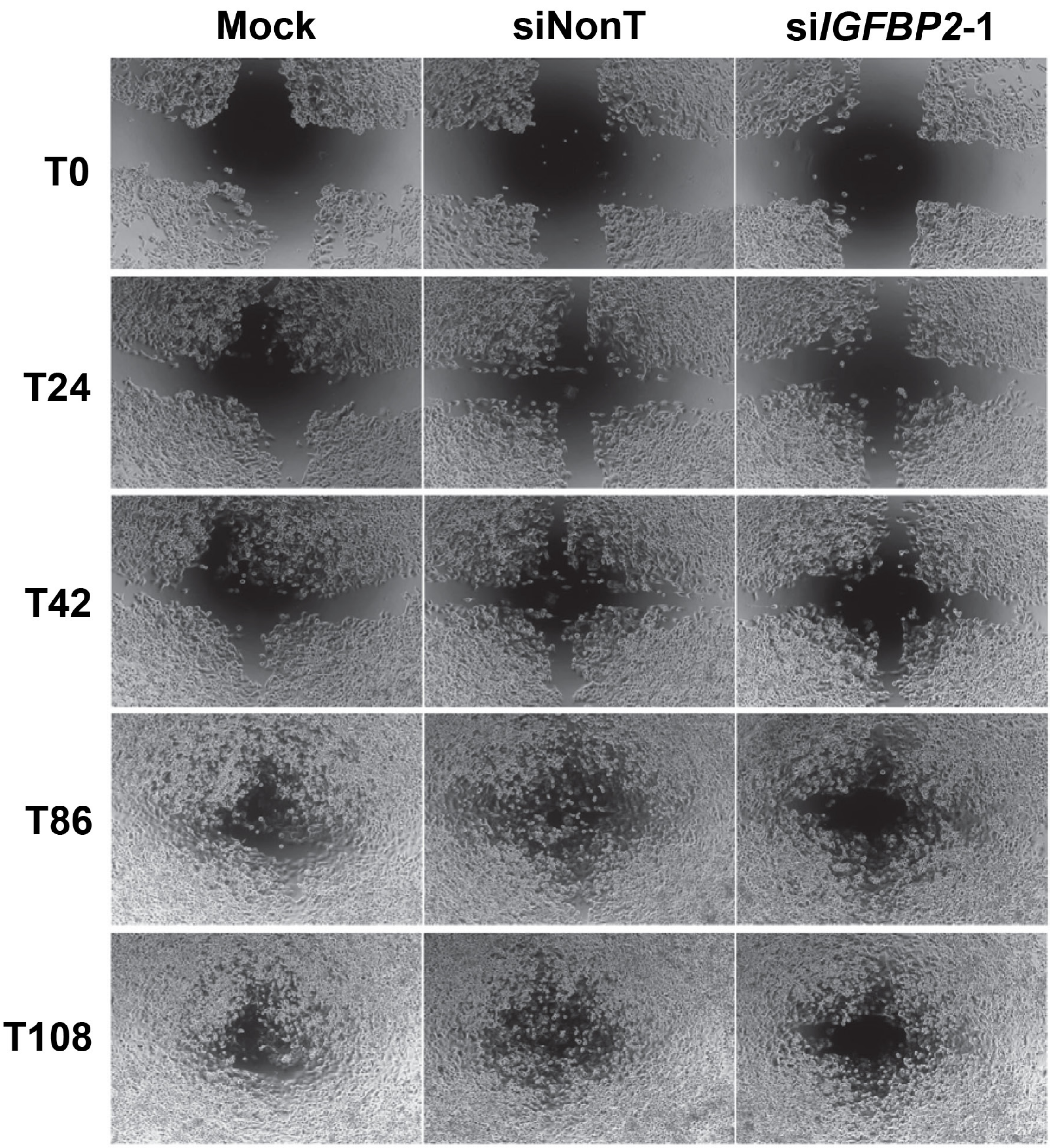

Supplementary Figure S6: (*Continued*) IGFBP2-mediated migratory and invasive capacity of EAC cells. B. mock-, siNon-targeting-, and si*IGFBP2*-treated Flo-1 cells were grown to confluence in 10% serum media, wounded with p20 pipette tips and periodically captured using a SPOT Idea 1.3MP camera for up to 20 hours and 108 hours, respectively, and measured using SPOT Basic software. Percent wound closure was calculated as 100–(T8/T0 × 100), where T8 represented the wound gap at 8 hours post-wound and T0 represented the original gap post-wound. Columns and error bars are the mean ± SD of 3 wells in each experiment.

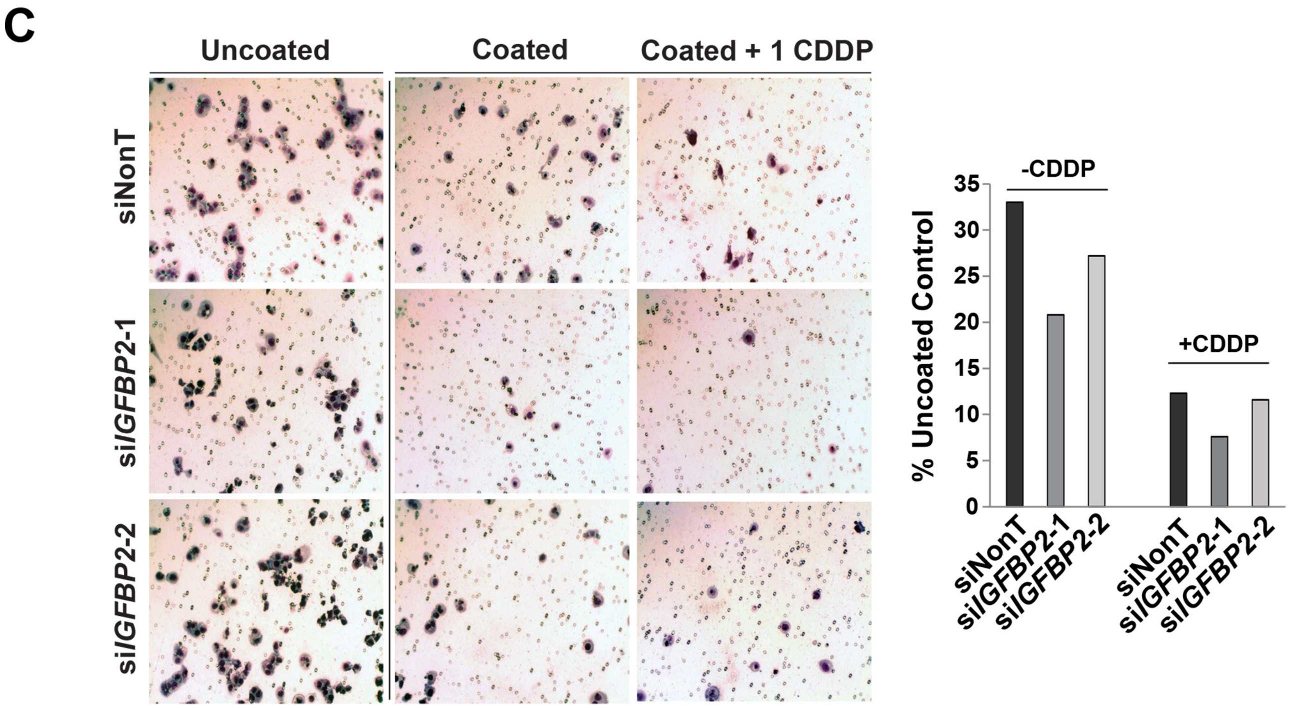

Supplementary Figure S6: (*Continued*) IGFBP2-mediated migratory and invasive capacity of EAC cells. C. Matrigel invasion was assayed for Flo-1 cells pretreated with individual ON-TARGETplus *IGFBP2* siRNAs or siNon-Targeting control, trypsinized and replated at 40, 000 cells per coated and uncoated transwell. Invaded cells were stained with Diff-Quick solution and imaged with a SPOT Idea 1.3MP camera. Percent invasion was calculated as $I_C/I_{UNC} \times 100$, where I_C represented the total number of invaded cells in 5 fields of each coated transwell and I_{UNC} represented the total number of invaded cells in 5 fields of the uncoated transwell for each treatment group.

A

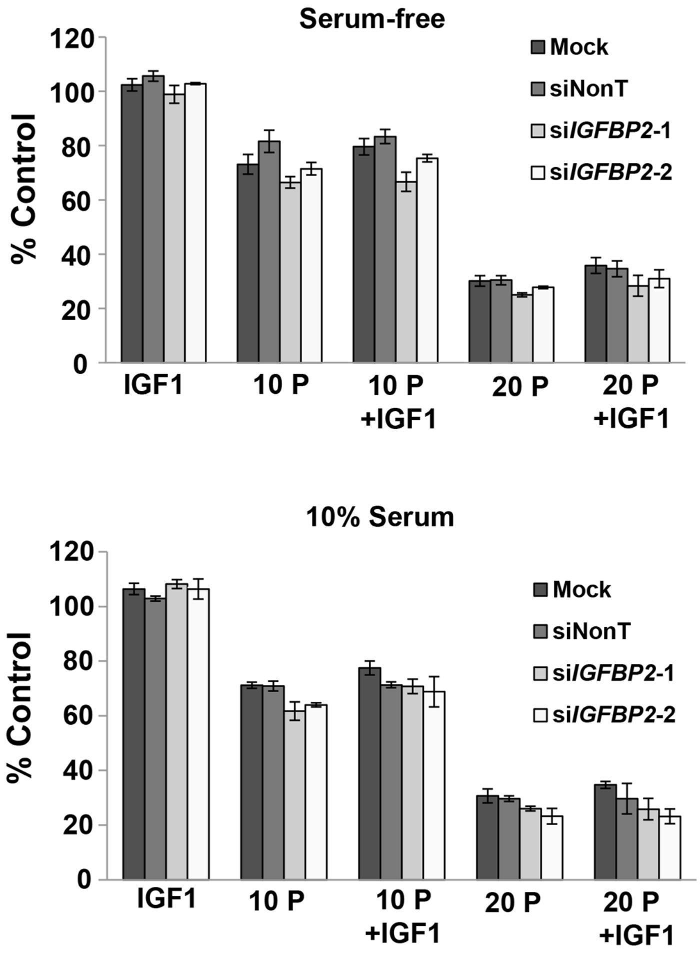

B

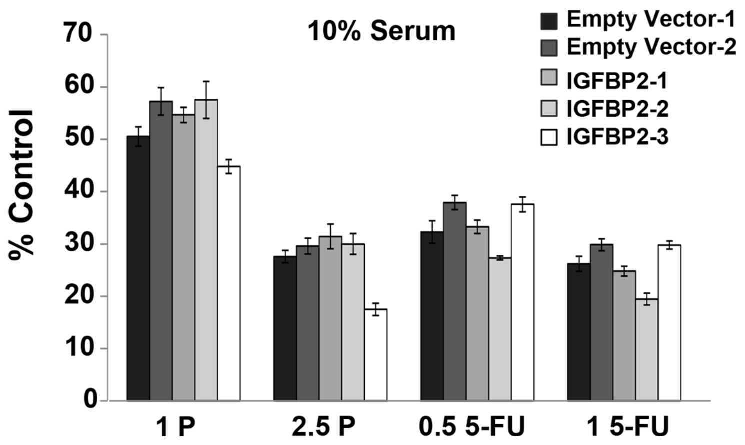

Supplementary Figure S7: Effect of IGFBP2 modulation on chemosensitivity in EAC cells. A. Following 24-hour treatment with individual ON-TARGETplus *IGFBP2* siRNAs, siNon-Targeting control or lipofectamine alone, Flo-1 cells were mock-pretreated or pretreated with 200 ng/mL IGF1 for 1 hour followed by mock-treatment or treatment with 10 or 20 µg/mL (33.3 µM or 66.7 µM) CDDP in serum-free or 10% serum DMEM for 24 hours. **B.** Empty Vector- and IGFBP2-OE33 clones were mock-treated or treated with 1 µg/mL (3.3 µM) CDDP, 2.5 µg/mL (8.3 µM) CDDP, 0.5 µg/mL (3.8 µM) 5-FU or 1 µg/mL (7.7 µM) 5-FU in 10% serum RPMI for 3 days. (*Continued*)

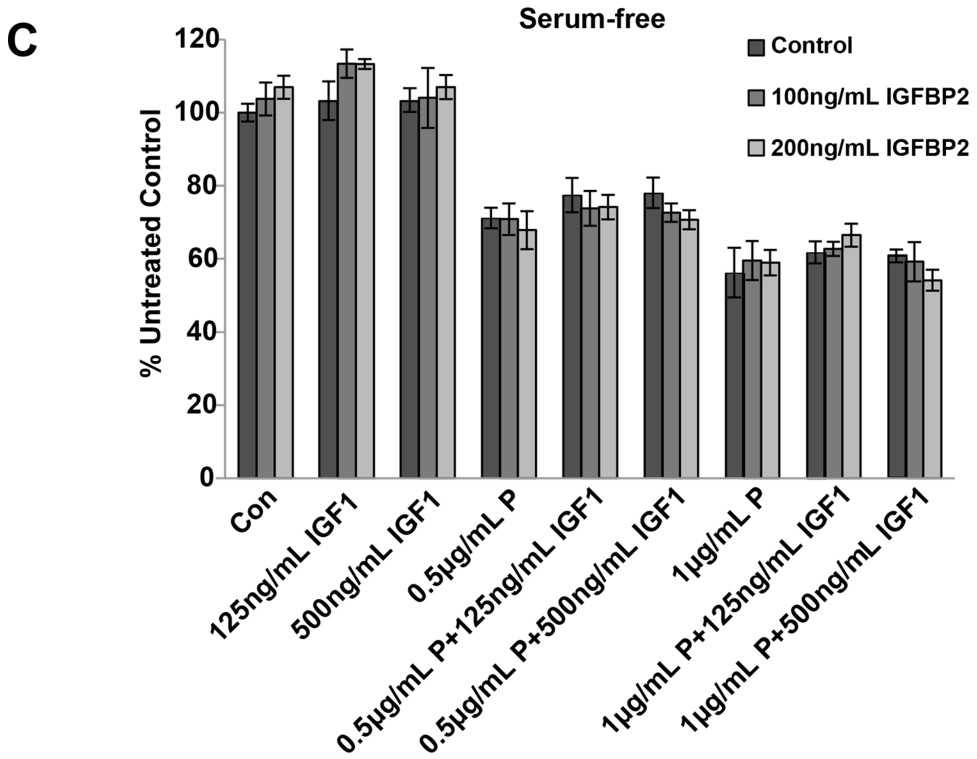

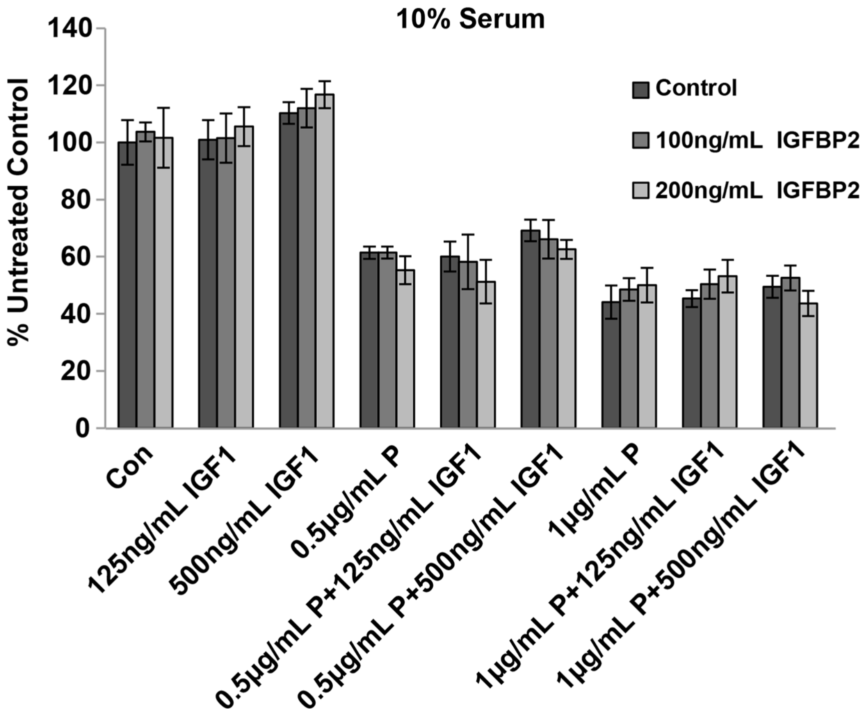

Supplementary Figure S7: (*Continued*) Effect of IGFBP2 modulation on chemosensitivity in EAC cells. C. OE33 cells were pretreated with PBS or 100 or 200 ng/mL human recombinant IGFBP2 for 30 minutes followed by addition of PBS or 125 or 500 ng/mL human recombinant IGF1 for 1 hour prior to 0.5 or 1 µg/mL (1.7 or 3.3 µM) CDDP treatment in serum-free or 10% serum RPMI for 3 days.

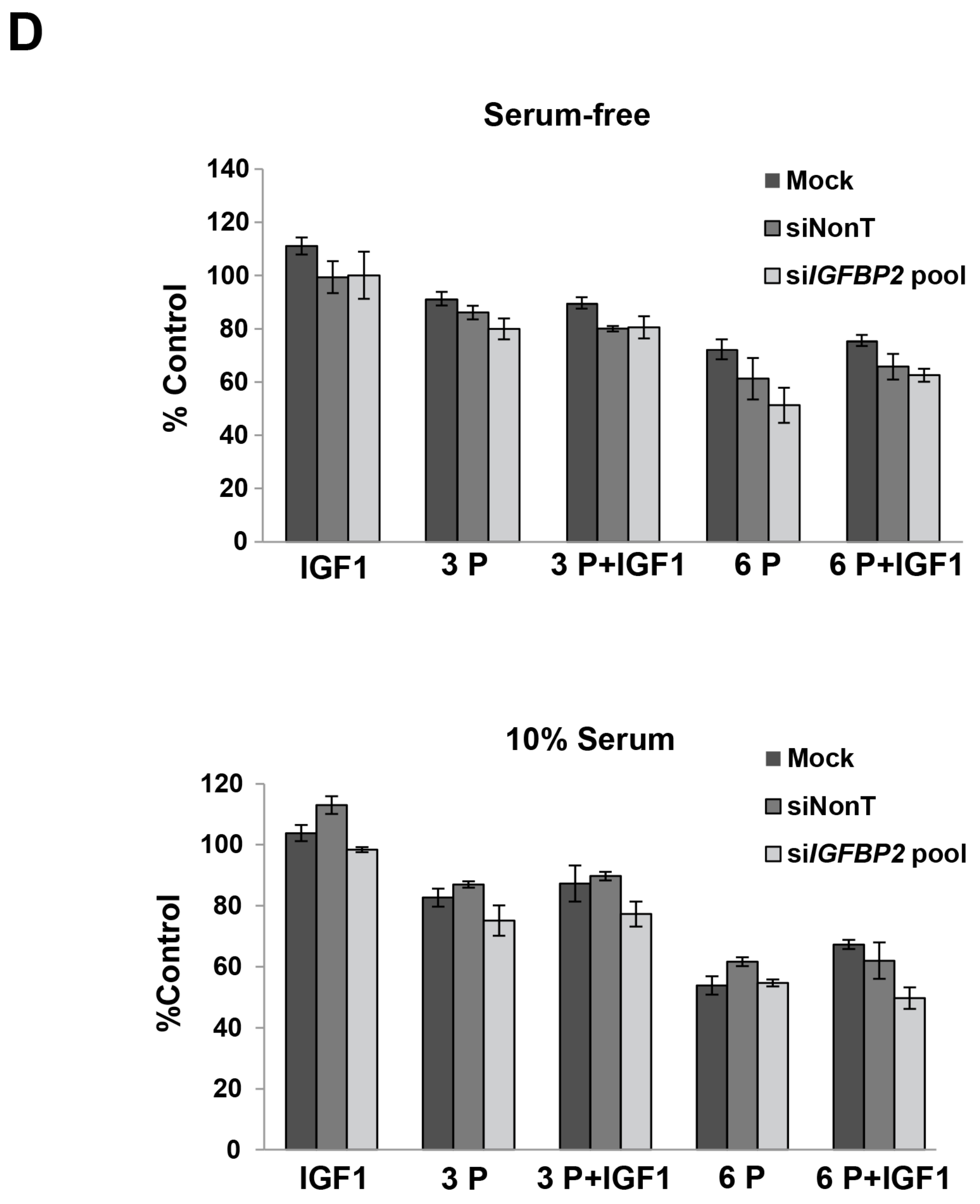

Supplementary Figure S7: (*Continued*) Effect of IGFBP2 modulation on chemosensitivity in EAC cells. D. Following 24-hour treatment with ON-TARGETplus *IGFBP2* siRNA SMARTpool, siNon-Targeting control or lipofectamine alone, OE19 cells were mock-pretreated or pretreated with 200 ng/mL IGF1 for 1 hour followed by mock-treatment or treatment with 3 or 6 µg/mL (10 or 20 µM) CDDP in serum-free or 10% serum RPMI for 3 days. WST analyses were performed to assess viability of all treated cells. Columns and error bars are the mean ± SD of 3 or more wells in each experiment. (10 P, 10 µg/mL CDDP; 20 P, 20 µg/mL CDDP; 1 P, 1 µg/mL CDDP; 2.5 P, 2.5 µg/mL CDDP; 0.5 5-FU, 0.5 µg/mL 5-FU; 1 5-FU, 1 µg/mL 5-FU; 3 P, 3 µg/mL CDDP; 6 P, 6 µg/mL CDDP).

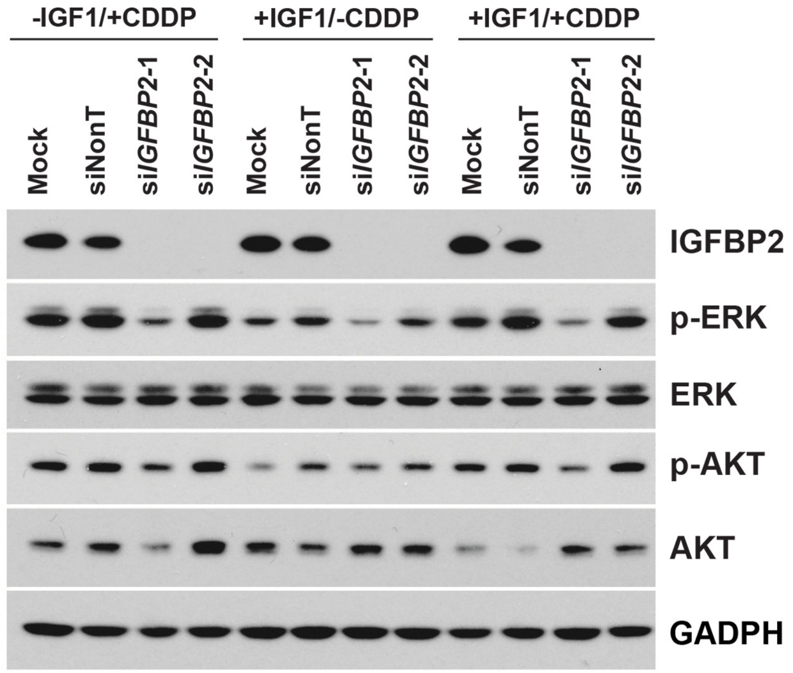

Supplementary Figure S8: Effect of IGFBP2 modulation on CDDP-induced signaling pathway activation in OE19 EAC cells. Following 24-hour treatment with individual ON-TARGETplus *IGFBP2* siRNAs, siNon-Targeting control, or lipofectamine alone, OE19 cells were mock-pretreated or pretreated with 200 ng/mL IGF1 for 1 hour followed by mock-treatment or treatment with 1 μg/mL (3.3 μM) CDDP in 10% FBS RPMI for 3 days. Protein lysates were collected, quantitated and analyzed by Western blot for IGFBP2, phospho- and total ERK, phospho- and total AKT and GAPDH expressions.

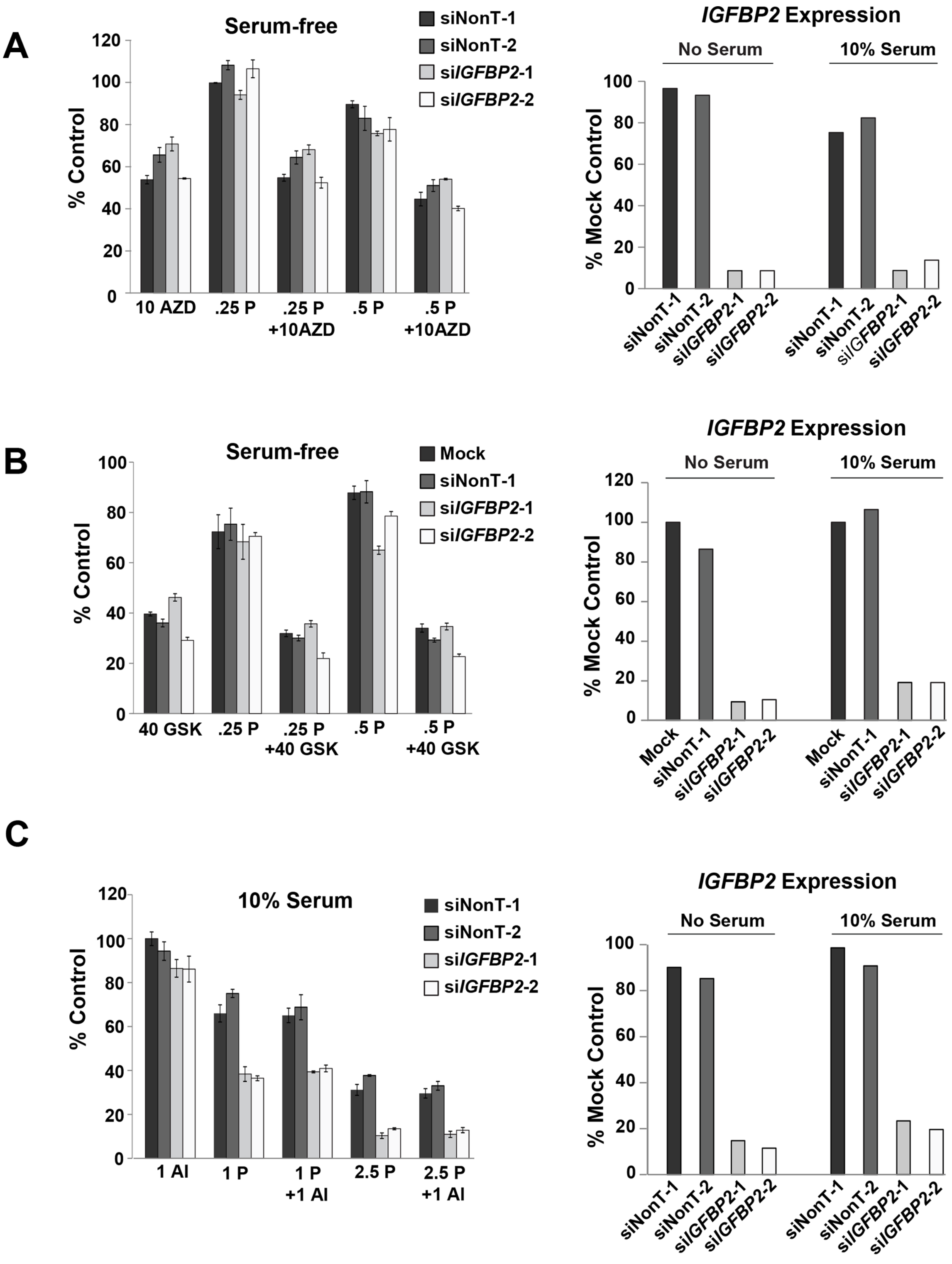

Supplementary Figure S9: Effect of small molecule inhibitors on si*IGFBP2*-mediated chemosensitization. Following 24-hour treatment with individual ON-TARGETplus *IGFBP2* siRNAs, siNon-Targeting controls or lipofectamine alone, Flo-1 cells were pretreated with 200 ng/mL IGF1 for 1 hour followed by mock-treatment or treatment with **A.** 10 µM AZD6244 (AZD), **B.** 40 nM GSK1120212 (GSK) or **C.** 1 µM AKT Inhibitor VIII (AI) and, lastly, mock-treatment or treatment with 0.25, 0.5, 1 or 2.5 µg/mL (0.8, 1.7, 3.3 or 8.3 µM) CDDP in serum-free (AZD and GSK) or 10% serum (AI) DMEM for 3 days. WST analyses were performed to assess viability of treated cells. Columns and error bars are the mean ± SD of 3 or more wells in each experiment. Concurrent real-time PCR was performed to verify IGFBP2 knockdown in all experiments. (10 AZD, 10 µM AZD6244; 40 GSK, 40 nM GSK1120212; 1 AI, 1 µM AKT Inhibitor VIII; .25 P, 0.25 µg/mL CDDP; .5 P, 0.5 µg/mL CDDP; 1 P, 1 µg/mL CDDP; 2.5 P, 2.5 µg/mL CDDP)

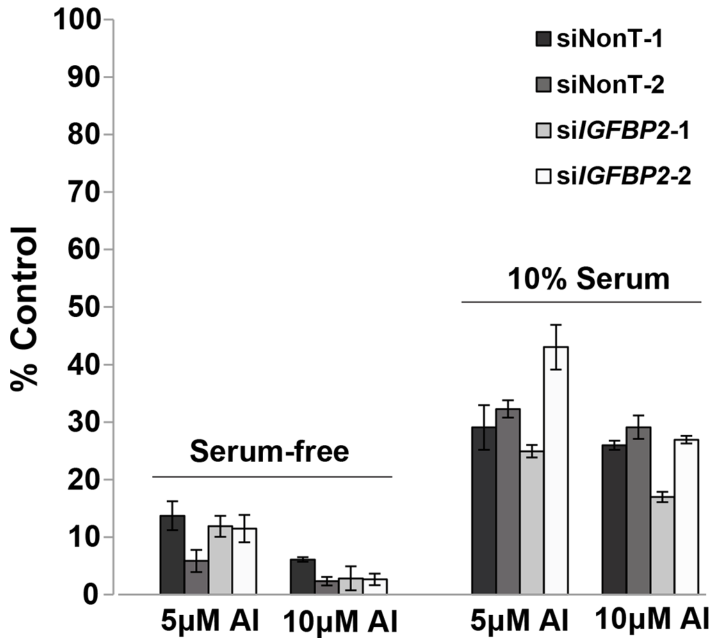

Supplementary Figure S10: Effect of complete inhibition of AKT with AKT Inhibitor VIII on si*IGFBP2*-treated Flo-1 EAC cells. Following 24-hour treatment with individual ON-TARGETplus *IGFBP2* siRNAs or siNon-Targeting controls, Flo-1 cells were mock-treated or treated with 5 or 10 µM AKT Inhibitor VIII in serum-free or 10% serum DMEM for 3 days. WST analyses were performed to assess viability of treated cells. Columns and error bars are the mean ± SD of 3 or more wells in each experiment.

Supplementary Table S1: Chemoradiation treatment regimens

Chemonaïve Tumors (n = 200)		Resistant Tumors (n = 16)		
**Adjuvant Therapy (n = 62)**	**Count**	**Neoadjuvant Therapy (n = 16)**	**Adjuvant Therapy (n = 5)**	**Count**
CDDP/5-FU	1	CDDP/5-FU/RT		1
CDDP/5-FU/RT	5	CDDP/5-FU/paclitaxel/RT		2
CDDP/5-FU/alpha interferon	1	CDDP/5-FU/RT	carboplatin/paclitaxel/RT; 5FU/ leucovorin/oxaliplatin*	1
CDDP/5-FU/alpha interferon/RT	1	CDDP/5-FU/RT	oxaliplatin/epirubicin/ capecitabine; 5-FU/ leucovoric/ oxaliplatin*	1
CDDP/5-FU/alpha interferon; RT*	2	CDDP/irinotecan/RT		1
CDDP/5-FU/alpha interferon; topotecan*	1	CDDP/paclitaxel/RT		1
CDDP/5FU/RT; docetaxel/carboplatin/ RT*	1	CDDP/etoposide/RT	oxaliplatin/docetaxel; carboplatin/capecitabine*	1
CDDP/RT; 5-FU/carboplatin/docetaxel*	1	CDDP/epirubicin/ capecitabine/RT	capecitabine	1
CDDP/gemcitabine; paclitaxel	1	Carboplatin/RT		1

(*continued*)

**Chemonaïve Tumors (*n* = 200)**		**Resistant Tumors (*n* = 16)**		
**Adjuvant Therapy (*n* = 62)**	**Count**	**Neoadjuvant Therapy (*n* = 16)**	**Adjuvant Therapy (*n* = 5)**	**Count**
CDDP/gemcitabine; paclitaxel*; 5-FU/ mitomycin C**	1	Carboplatin/paclitaxel/RT		3
CDDP/irinotecan/dexamethasone/RT	1	Carboplatin/paclitaxel/5-FU/RT		1
Carboplatin/5-FU/RT	2	Carboplatin/paclitaxel/RT	oxaliplatin/capecitabine	1
Carboplatin/paclitaxel	1	UNK/RT		1
Oxaliplatin/capecitabine/epirubicin	1			
5-FU/leucovorin/Eloxatin+/-GDC0449;Oxaliplatin/5-FU/ leucovorin*; paclitaxel/carboplatin/Herceptin*	1			
5-FU	1			
5-FU/RT	2			
5-FU/leucovorin/RT	5			
5-FU/mitomycin C	1			
5-FU/leucovorin; UNK*	1			
5-FU/leucovorin/RT; paclitaxel*	1			
5-FU/leucovorin/RT; paclitaxel/ carboplatin/RT*; oxaliplatin/irinotecan/ RT**	1			
5-FU/leucovorin/RT; oxaliplatin/ epirubicin/capecitabine*; capecitabine**	1			
Capecitabine/RT	2			
Docetaxel	1			
RT; docetaxel*; CDDP/5-FU**; mitomycin C***	1			
UNK	3			
UNK/RT	7			
UNK/RT; adriamycin/paclitaxel	1			
RT;UNK*	1			
RT	12			

Of the 216 patients examined in this study, 66 patients received some form of chemotherapy and 63 patients received radiation. Of those treated individuals, 15 patients received chemotherapy only, 12 patients received radiation only, and 51 patients received both chemotherapy and radiation. (UNK, unknown chemotherapy agent(s); RT, radiation therapy; *2nd line adjuvant therapy; **3rd line adjuvant therapy; ***4th line adjuvant therapy)

Supplementary Table S2: Primer sequences of genes examined by qRT-PCR

qRT-PCR Primers

Gene	Forward Primer (5′ to 3′)	Reverse Primer (5′ to 3′)	Reference
IGFBP2	AGCCCAAGAAGCTGCGACCAC	CTGCCCGTTCAGAGACATCTTGC	[24]
GAPDH	GTTCGACAGTCAGCCGCATCTTCT	AGGCGCCCAATACGACCAAATC	[65]
ACTB	ATGTGGCCGAGGACTTTGATT	AGTGGGGTGGCTTTTAGGATG	[65]
SNAI1	TATGCCGCGCTCTTTCCTCGT	GGTGGGCCTGGTCGTAGGG	
ZEB1	ACCCGCGGCGCAATAACG	CCTCCCTGGTAACACTGTCTGGTCTG	
VIM*	GCGATGGCCCAGCTGTAAGTTG	CTGCTGTCCCGCCGATTGAG	
MMP1	CCATTCTACTGATATCGGGGCTTTGA	CGATGGGCTGGACAGGATTTTG	
MMP9	GCGCTGGGCTTAGATCATTCCTCA	AGGCCGTGGCTCAGGTTCAGG	
SNAI2	AGATGCCGCGCTCCTTCCTG	AGTGATGGGGCTGTATGCTCCTGA	
FN1	AGAGGAGCACCACCCCAGACATTACT	CTCCAGGCCGGGACTCAGGTTAT	
CDH1	GACACCCGGGACAACGTTTATTACTATG	GGGAAGATACCGGGGGACACTCA	
CDH2	GGATGAAACGCCGGGATAAAGAAC	TGCTGCAGCTGGCTCAAGTCATAG	

Primers were designed using DNASTAR Primer Design Software as previously described [65]. PCR amplifications span introns to selective amplify RNA templates unless denoted by "*", which signifies a within-exon primer pair.
